# Gut contents, digestive half-lives and feeding state prediction in the soil predatory mite *Pergamasus longicornis* (Mesostigmata: Parasitidae)

**DOI:** 10.1007/s10493-017-0174-2

**Published:** 2017-09-01

**Authors:** Clive E. Bowman

**Affiliations:** Mathematical Institute, University of Oxford, Oxford, OX2 6GG UK

**Keywords:** Bayes, Compartmental modelling, Digestion, FIFO, Gut, Hysteresis, Input–output, Kinetics, LILO, Proteresis, Pulse-chase

## Abstract

Mid- and hind-gut lumenal changes are described in the free-living predatory soil mite *Pergamasus longicornis* (Berlese) from a time series of histological sections scored during and after feeding on fly larval prey. Three distinct types of tangible material are found in the lumen. Bayesian estimation of the change points in the states of the gut lumenal contents over time is made using a time-homogenous first order Markov model. Exponential processes within the gut exhibit ‘stiff’ dynamics. A lumen is present throughout the midgut from 5 min after the start of feeding as the gut rapidly expands. It peaks at about 21.5 h–1.5 days and persists post-feeding (even when the gut is contracted) up until fasting/starvation commences 10 days post start of feeding. The disappearance of the lumen commences 144 h after the start of feeding. Complete disappearance of the gut lumen may take 5–9 weeks from feeding commencing. Clear watery prey material arrives up to 10 min from the start of feeding, driving gut lumen expansion. Intracellular digestion triggered by maximum gut expansion is indicated. Detectable granular prey material appears in the lumen during the concentrative phase of coxal droplet production and, despite a noticeable collapse around 12 h, lasts in part for 52.5 h. Posterior midgut regions differ slightly from anterior regions in their main prey food dynamics being somewhat faster in processing yet being slightly delayed. Posterior regions are confirmed as Last-In-Last-Out depots, anterior regions confirmed as First-In-First-Out conveyor belt processes. Evidence for differential lability of prey fractions is found. A scheme is presented of granular imbibed prey material being first initially rapidly absorbed ($t_{\frac{1}{2}}$ = 23 min), and also being quickly partly converted to globular material extra-corporeally/extracellularly ($t_{\frac{1}{2}}$ = 36 min)—which then rapidly disappears ($t_{\frac{1}{2}}$ = 1.1 h, from a peak around 4 h). This is then followed by slow intracellular digestion ($t_{\frac{1}{2}}$ = 6.9 h) of the resultant resistant prey residue matching the slow rate of appearance of opaque pre-excretory egestive refractive grains (overall $t_{\frac{1}{2}}$ = 4.5 days). The latter confirmed latent ‘catabolic fraction’ (along with Malpighian tubule produced guanine crystals) drives rectal vesicle expansion as ‘faeces’ during the later phases of gut emptying/contraction. Catabolic half-lives are of the order of 6.3–7.8 h. Membraneous material is only present in the lumen of the gut in starving mites. No obvious peritrophic membrane was observed. The total feeding cycle time may be slightly over 52.5 h. Full clearance in the gut system of a single meal including egestive and excretory products may take up to 3 weeks. Independent corroborative photographs are included and with posterior predictive densities confirm the physiological sequence of ingestion/digestion, egestion, excretion, defecation, together with their timings. Visually dark midguts almost certainly indicate egestive refractive grains (xanthine?) production. Nomograms to diagnose the feeding state of *P. longicornis* in field samples are presented and show that the timing of these four phases in the wild could be inferred by scoring 10–12 mites out of a sample of 20. Suggestions to critically confirm or refute the conclusions are included.

## Introduction

**Fig. 1:**
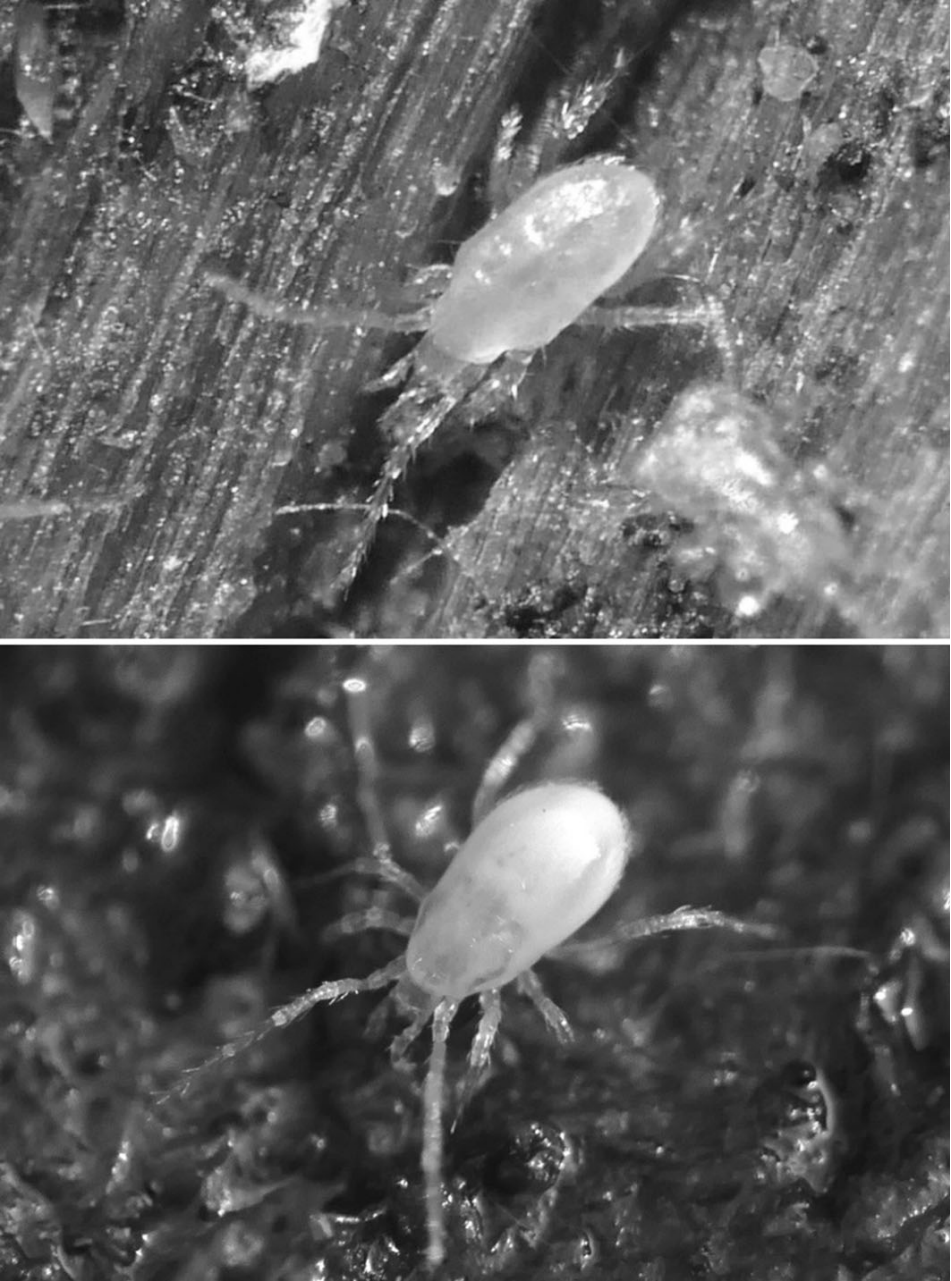
Typical soil predatory mesostigmatids in early stages of feeding/digestion. *Upper* Unfed. Despite integument transparency, no clear gut shape nor Malpighian tubules obvious. Matches *Pergamasus longicornis* $log_{e}$ values of up to 2.3 ($\equiv$ 0–10 min after start of feeding; see Fig. 13 and Bowman 2017). From a colour photograph, 29 October 2015. *Lower* Soon after stopping feeding. Note here the pale gut centrally in the idiosoma replete with imbibed prey material but with the lack of white crystalline guanine in the posterolateral Malpighian tubules. Matches *P. longicornis* $log_{e}$ values of 2.3–5.5 ($\equiv$10 min–4 h after start of feeding; see Fig. 13 and Bowman 2017). From a colour photograph, 31 October 2015. Both photos: 1.5 mm;  Germany, Schleswig-Holstein, Mohrkirch garden, Brache, log pile ©Lennart Bendixen with permission

There is the opportunity in any animal that ingests food, for digestion to occur within the gut before nutrient assimilation. This is especially true of poikilotherms, many of which may have very extended periods between consuming a large meal. Indeed in forest soils, opportunities to feed may come rarely for small predators such as mesostigmatid mites (Fig. 1). Amongst the free-living parasitids, mites of the genus *Pergamasus* (see Witalinski 1971) are both large and striking to the eye in leaf litter (being rapidly moving hunters around 1 mm in size). Bowman (1984)) states, without detailed justification, that the length of digestion in the predatory soil mesostigmatid *Pergamasus longicornis* (Berlese) (Parasitidae) was approximately 1 week in the laboratory. Using gut expansion/contraction measures, Bowman (2014) however, better estimated total feeding and digestion time in this poikilotherm in the laboratory as 52.5 h. These range of figures agree with the complete disappearance of a meal in 3–7 days found on examination of a slightly smaller mesostigmatid *Echinolaelaps echidninus* by Kanungo (1964). However, Bowman (2017) in following the time for a pulse of larval dipteran prey to be fully processed in *P. longicornis* into Malpighian waste guanine crystals (which takes up to 10 days post the commencement of feeding), points out that visible gut expansion/contraction is not a comprehensive measure of all nutritional processes. Examination of gut lumen contents is crucial in determining what a mite actually consumes, and how it copes with starvation. It was only in this way that *Riccardoella limacum* (Acari: Trombidiformes) was shown to actually be a blood-feeder of pulmonate molluscs and not a mucophage (see Baker 1970). Examining lumenal changes over time too, identified ‘tick-grown’ haemoglobin cystals as a nutrient reserve in the argasid *Ornithodorus moubata* (Smit et al. 1977), and how oribatids survive adversity (Hubert and Šustr 2001; Smrž 2002). Bowman (2017) also pointed out that an understanding of the time course of the production of any waste material in the gut lumen would help confirm or refute explanations of pergamasid digestive mechanisms offered. Further he described potential evidence for a hidden ‘egestive fraction’ of partly assimilated-partly catabolised material driving mesostigmatid rectal vesicle expansion. Irrespective of whether this arises from intra- or extracellular digestion, such pre-excretory material ought to be detectable in the gut lumen during feeding on prey by *P. longicornis* if systematically looked for.

Feeding and digestion in the soil predatory mite *P. longicornis* can be conveniently divided into certain key phenomena (see Bowman 2014, 2017):-

- rapid gut filling by prey fluid imbibition (with concomitant salivary processes);
- continued gut filling by imbibition (with the concentration of gut contents through fluid loss via coxal droplets);
- slow gut emptying through prey digestion;
- subsequent catabolism and excretory product appearance (and waste voiding).

This comprehensive paper, as part of a wide ranging investigation of pergamasid digestive physiology is based upon detailed serial histological data scored from a temporal series of this mite at various times up to 14 days from the onset of feeding. The mite’s physiology is expected to be stiff—the half-life of excretion of 53 h reported by Bowman (2017) is the same order of magnitude as the reported total feeding and digestion time (52.5 h—see Bowman 2014). The basic concept of this study is that the lumen and the contents of the gut lumen should change over time as digestion proceeds i.e. architecture should inform process. Digestion in parasitic or predatory mites is assumed to be continuous (Lindquist et al. 1996) so, lumenal contents that are highly correlated over time and sharing appropriate time constants and lags indicate possible transitions in imbibed prey material. Consequently this paper seeks:-

- to describe the gross changes in the lumen and lumenal contents of the gut including any possible pre-excretory products that are present during feeding and digestion,
- to examine their temporal flow, and
- to look for contra-evidence to the above four-part scheme.

A critical synthesis of all of the lumenal changes is offered together with corollaries and predictions to confirm or refute the mechanisms involved.

Stiff dynamic systems like in pergamasid physiology exhibit sub-processes that vary markedly in their rate constants by orders of magnitude. The underlying experimental concept of this paper is that the asynchrony of gut lumenal changes and the relative magnitudes of the kinetic summaries of gut contents will inform the inference of what metabolic transitions are happening and facilitate the unpicking of the overall processes occurring in the mite’s gut system. In other words, process should inform physiological function. A variety of analytical approaches (including Bayesian segmentation and compartmental modelling) are used to solve this inverse problem and yield biological insights. The first order differential equation linear system model of Bowman (2017) is assumed.

Based upon previous tick physiological results, Bowman (2014) gave three alternatives for the control of the commencement of digestion in *P. longicornis*. He also suggested gut elimination of food may have different kinetics posteriorly compared to anterior gut regions. Later Bowman (2017), in describing excretory processes in this mite, inferred the presence of a significant hidden catabolic ‘egestive’ fraction in the gut. None of these assertions have yet been critically challenged. Explicitly testing them yields the following specific hypotheses (accordingly numbered below for cross-reference to the Discussion):-

(i) Is digestion triggered by maximal midgut stretching—estimated as 3.5 min post start of feeding? or
(ii) Is digestion triggered by maximum midgut expansion—estimated at 10 min post start of feeding? or
(iii) Is digestion triggered by stopping (the act of) feeding—estimated at 56–96 min after the start of feeding?
(iv) Is food elimination slower in the posterior gut compared to that anteriorly?
(v) Is there any evidence for a rapid, run-away breakdown or catabolism suitable to match the observed input into the occurrence of guanine crystals in the Malpighian tubule system?
(vi) Is there evidence of an ‘egestive’ intermediate in the lumen with appropriate catabolic dynamics?
  Does it occur from about 4–8 h through to 18 h?
  Does it occur out to 48 h post-commencement of feeding when excretory guanine appears in the Malpighian tubules?
  Could it be the midgut-borne precursor or catabolic surrogate of Malpighian tubule guanine? and
  Is the egestion (*E*) half-life of the order of the catabolic (C) half-life (as critically needed for Bowman (2017)’s system physiological explanation)?
  Is the total feeding cycle time (ingestion, digestion, egestion) 52.5 h or not? and
  Does an egestive phase reconcile the difference between 52.5 h and 1 week (claimed by Bowman (1984) without justification) for the total feeding cycle time, and better match the figures of Reichle and Crossley (1965)?
(vii) Does indigestible membraneous or faecal material exist in the gut lumen especially late on after feeding, and if so is it mostly in the rectal vesicle?
(viii) Is there confirmatory evidence for hunger/starvation in *P. longicornis* commencing around 10 days and complete idiosomal clearance of material from a single larval dipteran prey by 15 days post-prandially?

Answering these eight questions will confirm or refute the mechanisms previously posed.

Once the resultant overall scheme of gut changes and function is crystallised by detailed critical argument, its consilience will be assessed using independent photographic evidence. Finally, physiological function should inform life-style adaptation (see Croft et al. 2004). So, the ordered schema will then be applied to field diagnosis predicting the likely feeding state of pergamasids in the wild.

## Materials and methods

**Fig. 2:**
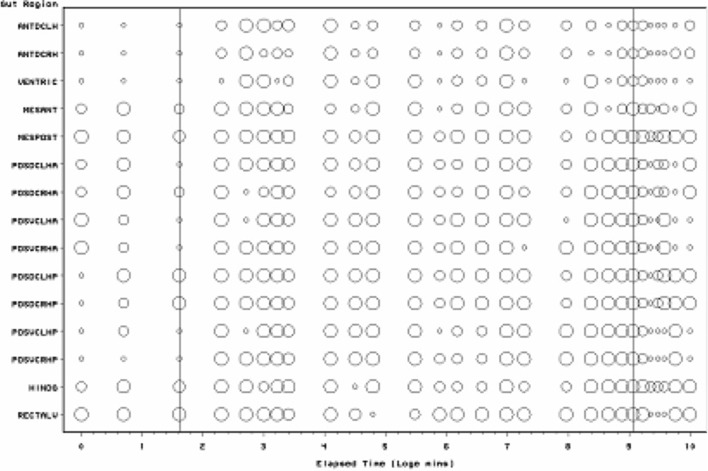
Schematic of *Pergamasus longicornis* lumen status for each gut region (ordered anterior to posterior) as: Present (*large circles*), or, Absent (*small dots*). *Intermediate size circles* represent ambiguity over replicates. Time is from the commencement of feeding and is on a natural logarithmic scale. *Grey lines* are at the 5 min and 8640 min (=144 h) after the commencement of feeding Bayesian posterior estimates of the change points overall. These mark first a very rapid appearance in lumenal presence then a disappearance, respectively. Associated gut region abbreviations:- Anterodorsal caecum LH = ANTDCLH; Anterodorsal caecum RH = ANTDCRH; Ventriculus = VENTRIC; Mesenteron anterior = MESANT; Mesenteron posterior = MESPOST; Posterodorsal caecum LH anterior = POSDCLHA; Posterodorsal caecum RH anterior = POSDCRHA; Posteroventral caecum LH anterior = POSVCLHA; Posteroventral caecum RH anterior = POSVCRHA; Posterodorsal caecum LH posterior = POSDCLHP; Posterodorsal caecum RH posterior = POSDCRHP; Posteroventral caecum LH posterior = POSVCLHP; Posteroventral caecum RH posterior = POSVCRHP; Hind gut = HINDG; Rectal vesicle = RECTALV

*Pergamsus longicornis* mites were collected by hand from leaf litter sampled at a variety of deciduous woodland sites in Merseyside and Hertfordshire, UK in 1977. Mites were kept individually at room temperature and >90% rh throughout. Mites were starved for 1 week and then fed one final instar larva of the fruit fly *Drosophila melanogaster *(vestigial wing strain). At 28 distinct pre-specified log-spaced elapsed times from the commencement of feeding, a total of thirty four mites were destructively fixed in cold Susa, dehydrated through graded isopropyl alcohol, into xylene and double embedded in celloidin and paraffin wax. Sections were taken at 7$$\mu$$, stained with Mallory’s Triple Stain and mounted in DePeX. Lumenal contents in each of 15 mid- and hind-gut regions (for abbreviations see Fig. 2) were characterised temporally with a three point score as:- Granular (Lots = 2, Some = 1, None = 0); Globular (Lots = 2, Some = 1, None = 0); Membranous (Lots = 2, Some = 1, None = 0); Other (Lots = 2, Some = 1, None = 0). No attempt was made in this analysis to utilise any colour changes in the stained lumenal contents over time. Whether the gut lumen contained tiny refractive granular material under Nomarski phase contrast interference light microscopy in each region was also scored (Lots = 2, Some = 1, None = 0). The grains of this material were an order of magnitude smaller than the large guanine crystals observed within Malpighian tubules (see Bowman 2017). Nor were they as refractive. Nor noticeably birefringent. Relative occurrence of scored material is assumed to measure relative amounts. Amounts were not normalised by gut volume to derive a surrogate of concentration. Due to collecting constraints, no distinction was made between male (elapsed time after the start of feeding: 0, 2, 5, 5, 10, 25, 60, 90 min, 6, 12, 48, 96, 168, 192, 240, 288 h) and female (0, 15, 20, 20, 30, 30, 60, 90 min, 2, 4, 8, 18, 24, 72, 120, 144, 216, 336 h) mites. Genders are pooled as in Bowman (2014). All data were coded, stored and manipulated in Access97, Excel, or SAS6.12. Diagrams were produced in SAS 6.12, Excel, and Powerpoint. Elapsed time from zero was log transformed (time > 0). A logarithmic scale for elapsed time was chosen upon the simplest assumption that physiological changes represent first order rate processes (Lister et al. 1988)—this is the same assumption as in the models of Sabelis (1990). Natural (log$$_{e})$$ logarithms were used throughout.

Bayesian modelling of the probability (*P*) of a lumen or specific lumenal contents used OpenBugs 3.23 with non-informative Jeffreys priors (Congdon 2001). A burn-in of 30,000 updates followed by summaries of a further 30,000 updates was used. The notation ‘hat’ is used to mean ‘estimated value’. The log odds ratio comparing anterior sections (subscript = 1) to posterior sections (subscript = 2) was calculated as $ln[\frac{\pi _1}{(1-\pi _1)}\cdot \frac{(1-\pi _2)}{\pi _2}]$ where $\pi$ is the estimated probability of the occurrence of lumen or specific lumenal materials in the gut. This statistic is asymptotically Normally distributed as $N(ln(odds\ ratio),\sigma ^{2}_{ln(odds\ ratio)})$ under the null of no distinction between the two regions. A two-sided *z*-test is used herein. Posterior predictive distributions (Aitchison and Dunsmore 1975) used non-informative *Beta*(0.5, 0.5) or $Dirichelet(\alpha \ concentration=1)$ parameter priors and a uniform prior over the time points for segmentation (see Congdon 2001) with a burn-in of 30,000 updates followed by summaries of a further 1000 updates in OpenBugs 3.23.

### Change-point estimation

Bayesian change-point modelling was carried out in WinBugs 1.3. Rather than ‘eye-balling’ results to look for temporal differences, a two segment continuous ‘broken stick’ first-order time-homogeneous Markov model was used to divide up the results (for all gut regions including the rectal vesicle together) either side of a putative physiological change-point between two processes as follows:- Let $y_{i,j}$ be the state (out of $k=3$ states: 0,1,2) for the *i*th ($i=1$ to 15) gut region at the *j*th ($j=2$ to 34) elapsed time point. Then, here, $y_{i,j}\sim Multinomial(p_{Q,i}*y_{i,(j-1)}$) where $y_{i,(j-1)}$ is the state (out of $k=3$ states: 0,1,2) for the *i*th ($i=1$ to 15) gut region at the *previous* (i.e. ($j-1$)th, $j=1$ to 33) elapsed time point. $p_{Q,i}$ is then a (3 × 3) matrix of state transition probabilities for the *i*th region between time point ($j-1$) and *j* (i.e. it is a first-order Markov process). *Q* is an indicator variable [$Q=1$ or 2] showing if that time point (*j*) is to the left or the right of the position of a continuous change point between two distinct processes and thus which of two ($3 \times 3$) transition matrices (i.e. $p_{1,i}$ for before, or $p_{2,i}$ for after) to employ. Treating the gut as a homogeneous unit over *i* regions (i.e. assuming gut regions were independent replicates of each other) is the same as setting $p_{Q,i}=p_{Q}\ \forall \ i$. A time-heterogeneous process would allow the elements of $p_{Q}$ to vary with *j* for all *j*. The underlying assumption is that lumenal contents (prey material) remain broadly the same type in appearance and micro-evolution until they are all effectively converted to another type, so that the change point indicates an overall macro-evolution in the imbibed prey material within the gut. The order of estimates across lumenal content type informs any inter-conversions proposed.

A vague normal distribution, *N*(0, 100), was logged and used as a convenient distribution to generate prior transition probabilities following (Congdon 2001). Sensible initial values were given to Winbugs 1.3. This stochastic model ignores the repeated nature of measuring multiple gut regions within individual mites for simplicity of estimation given the limited data. No data was used more than once except that a continuity through the change point was allowed as there was a need for a smooth constraint i.e. the two processes were to be modelled simultaneously. The change point was given a uniform multinomial prior distribution. This model ignores the lack of exactly spaced (log) time intervals and the replicated nature of some of the time points in this study (the latter were averaged and the resultant re-scored as closest to 0, 1, or 2 before change-point estimation). The two matrices (one before the change-point, one after) are ‘nuisance variables’ i.e. a means to an end, to best estimate the phase-change point—not an end in themselves. Accordingly, no estimates of the transition matrices are reported herein, rather $p_{Q}$ should be considered as only indicative, but not definitive, of the detailed micro-physiological processes occurring. One thousand iterations of the Gibbs sampler were used for ‘burn in’ to empirical stability of estimates and discarded. Bayesian posterior distributions for the change point given the priors and the observed data were estimated from a further 1000 samples and summarised.

This artificial segmentation was employed for the convenient estimation of the characteristics of two phases although it assumed that the underlying biological processes occur contemporaneously to an extent. This simplicity is assumed as using special time points for physiological processes to be switched on or off is a more complicated model only justifiable if there is evidence that it is necessary to explain the results with a discontinuous threshold (Occam’s Razor applies!). The resultant change point (or phase-change) estimated represents the time when an important empirical change in the lumenal contents results occurs. A sensitivity analysis (*details not shown*) for the Bayesian estimate of the location of the change point (for all data bar that scored as ‘Membraneous’ or ‘Other’) was undertaken by restricting the prior distribution of the change point to be multinomial zero up to elapsed time *j* for all values of *j* = 1 to 34 and examining the summarised estimates.

### Kinetic estimation

Kinetic modelling of the Bayesian posterior mean estimates was done by exponential stripping (Kirkup and Sutherland 1988) on an arithmetic time scale using Excel2011. Kinetic modelling excluded the ectodermic rectal vesicle as a part of the gut. A first order differential empirical model for the probability (*P*) of a lumen or specific lumenal contents occurrence at any time *t* in the observed gut ‘compartment’ of interest was used via the *directed* output input relationship:-

$$\begin{aligned} P[t]=a_{el}.e^{b_{el}.t}-a_{in}.e^{b_{in}.t} \end{aligned}$$

where the subscript *in* denotes input, and the subscript *el* denotes elimination ( $\equiv$ output). A gut ‘compartment’ of interest here could be the lumen itself or could be a type of lumenal content e.g. ‘globular’ material. For a rate constant $b_{el}<0$ the effective size of $a_{el}$ declines away with a half-life $t_{\frac{1}{2}}=\frac{ln(2)}{b_{el}}$. If there was no input then $a_{el}=P[0]$. The stripping process traditionally fits late occurring data under the assumption that elimination is dominating and that absorption ( $\equiv$ input) has essentially completed. Then subtracts the fitted values (assuming this elimination) from the original data to form adjusted data. Then fits the early occurring adjusted data when input is dominating and elimination only just under-way in order to estimate the input (absorption) parameters. So, the contribution $a_{in}.e^{b_{in}.t}$ represents in a positive sense the first order ‘elimination’ ($b_{in}<0$) from a notional previous or up-stream compartment being now input into the observed down-stream gut compartment of interest. In this example $\hat{a}_{in}=\hat{a}_{el}=e^{(max[\hat{P}_{t}])}$ since stripping should ensure a crossing or knot point for the functions at the peak of *P*[*t*]. The half-life of the input function is $\frac{ln(2)}{b_{in}}$. All physiological phenomena are assumed to be present from $t=0$ after the start of feeding, so all other matters being equal, the linear superposition principle ensures that the shape of any profile from gut compartment to compartment spreads out as the ‘food signal’ passes along a series of compartments arranged one-after-another. The initial signal thus becomes ‘blurred’ and attenuated (*per force* diminished in extent)—even if an interlinked network of compartments are assumed. For the purposes of illustration, in some instances, input models assuming no elimination were estimated directly from the rise in unadjusted data over specific time periods and through particular maximum values. This simpler approach is appropriate when the half-lives of absorption and elimination are different by orders of magnitude.

## Results

Scoring for the presence of a lumen in each mid- and hind-gut region of *P. longicornis* is presented in Fig. 2. The Bayesian posterior estimates of the change points overall are at 5 min and 8640 min (=144 h) after the commencement of feeding. These mark first a very rapid appearance in lumenal presence then a disappearance, respectively. Initially, the gut is contracted with no lumen. There is a very rapid appearance of a lumen within the gut as it expands (by 5 min) which persists until at least 144 h after the pergamasid commences feeding. There is no evidence of regurgitation and loss of lumen during early feeding in this data.

**Fig. 3:**
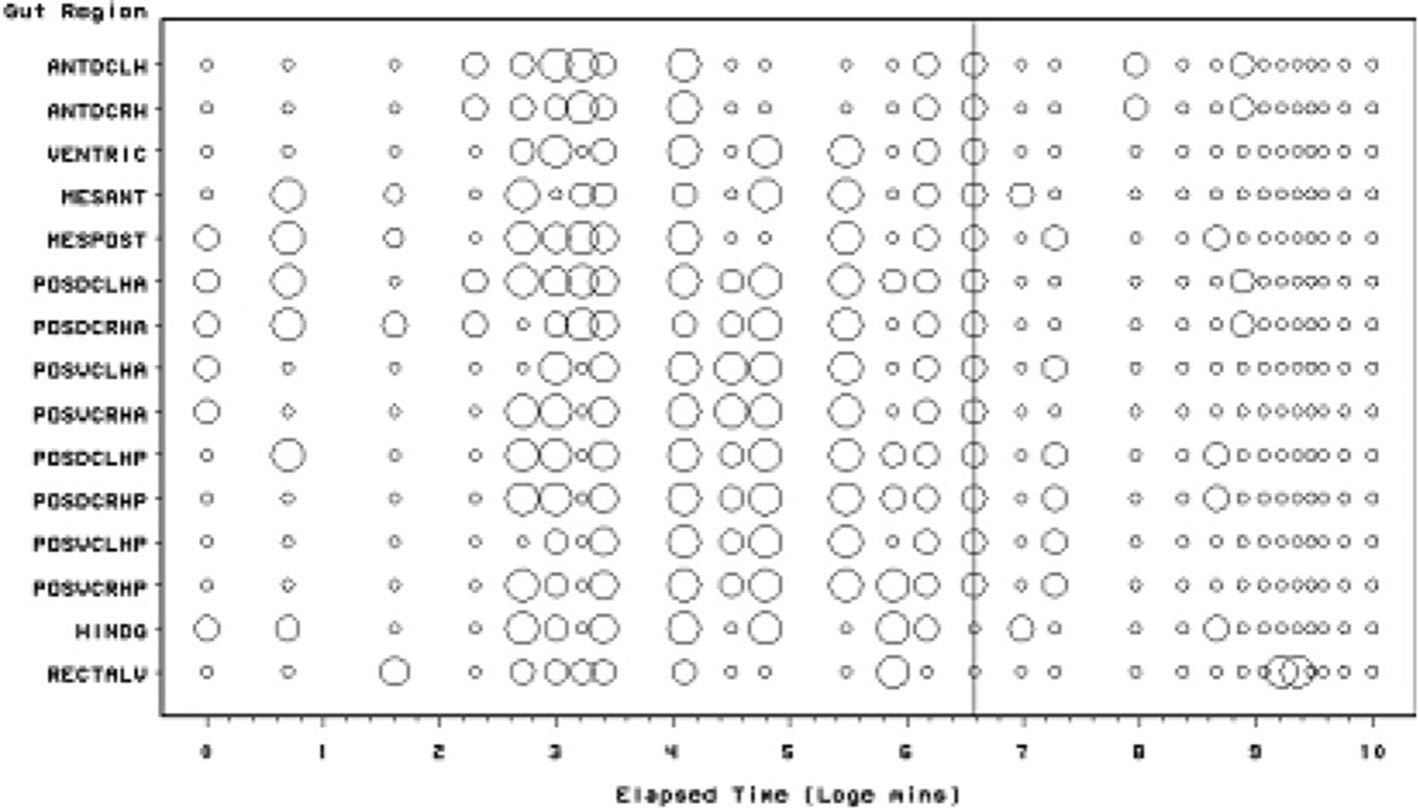
Schematic of *Pergamasus longicornis* lumenal (non-refractive) granular material in each gut region (ordered anterior to posterior) as: Lots (*large circles*), or, None (*small dots*). *Intermediate size circles* represent presence of some granular material or an average over replicates. Time is from the commencement of feeding and is on a natural logarithmic scale. *Grey line* is at the 720 min (=12 h after the commencement of feeding) Bayesian posterior estimate of the change point overall. This marks the start of a noticeable collapse in the presence of lumenal granular material—all ingested food material essentially disappearing after this point

Scoring of each gut region for the presence of lumenal (non-refractive) granular material is presented in Fig. 3. The Bayesian posterior estimate of the change point overall is at 720 min (=12 h after the commencement of feeding). This marks the start of a noticeable collapse in the presence of lumenal granular material—all ingested food material essentially disappearing after this point. Granular material appears quickly but initially sporadically within the caeca (in 2 min—follow-up work may show perhaps as fast as the 2 s reported for scorpions see Alexander 1972). This is slightly faster than the 3–5 min for the saprophagous mite *Rhizoglyphus echinopus* (Akimov 1971). It fits in with the highspeed feeding observations by Flechtmann and McMurtry (1992) on the phytoseiid predators *Galendromus occidentalis* and *Amblyseius similoides*. Twelve hours post the commencement of feeding marks the abrupt transition from the presence of imbibed granular material in the mid- and hindgut lumen to its absence. This broadly matches the commencement of initial gut emptying across all gut regions estimated by Bowman (2014) as 9.5–15.5 h after the start of feeding.

**Fig. 4:**
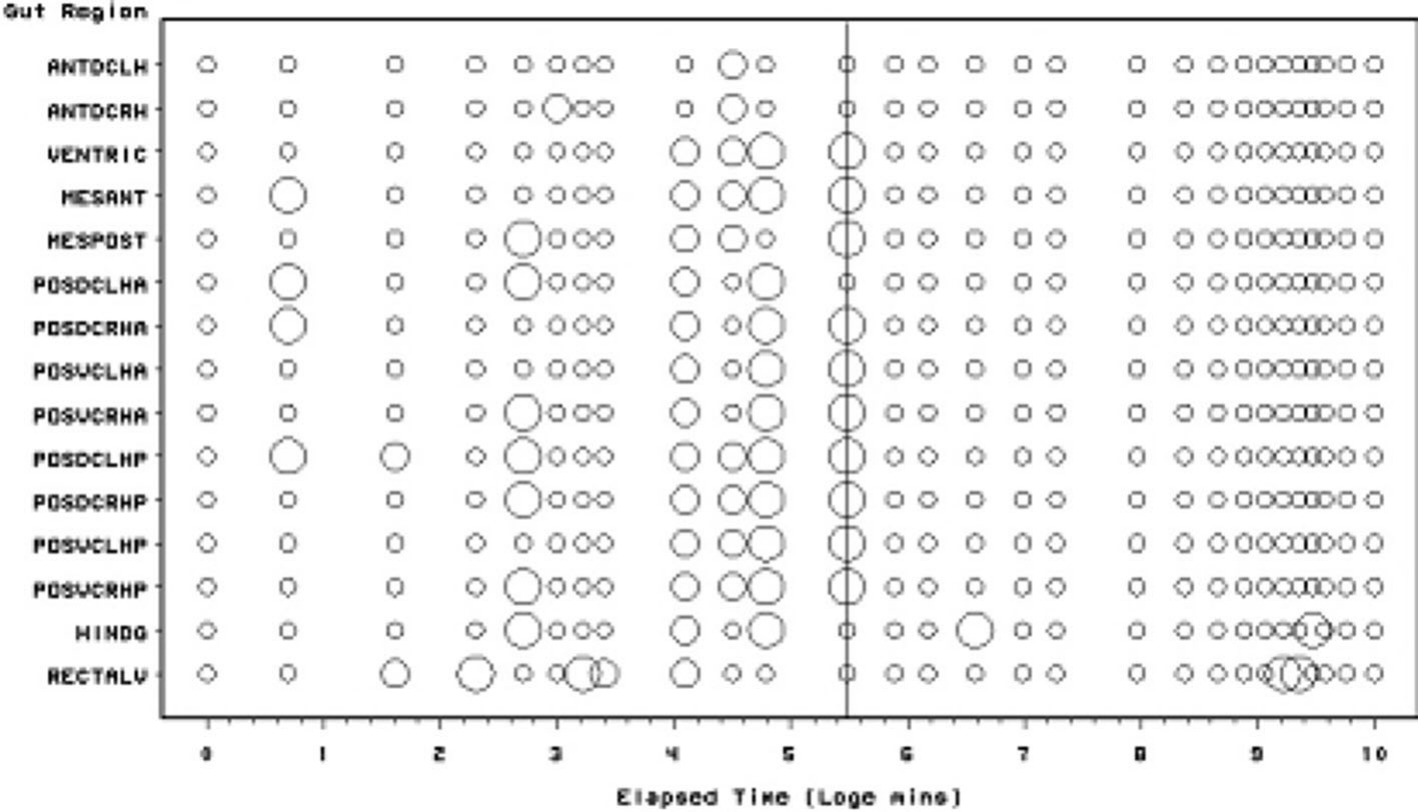
Schematic of *Pergamasus longicornis* lumenal globular material in each gut region (ordered anterior to posterior) as: Lots (*large circles*), or, None (*small dots*). *Intermediate size circles* represent presence of some globular material or an average over replicates. Time is from the commencement of feeding and is on a natural logarithmic scale. *Grey line* is at the 240 min (=4 h after the commencement of feeding) Bayesian posterior estimate of the change point overall. This marks a noticeable decline in the presence of lumenal globular material

Scoring of each gut region for the presence of lumenal globular material is presented in Fig. 4. The Bayesian posterior estimate of the change point overall is at 240 min (=4 h after the commencement of feeding). This marks a noticeable decline in the presence of lumenal globular material. Globular material is the second type of tangible prey material appearing histologically in the gut lumen over time. It is present sporadically during feeding but only consistently occurs as a discrete pulse after about 1 h (i.e. after the cessation of feeding on the prey at 56–96 min—Bowman 1987). It vanishes abruptly after 4 h.

**Fig. 5:**
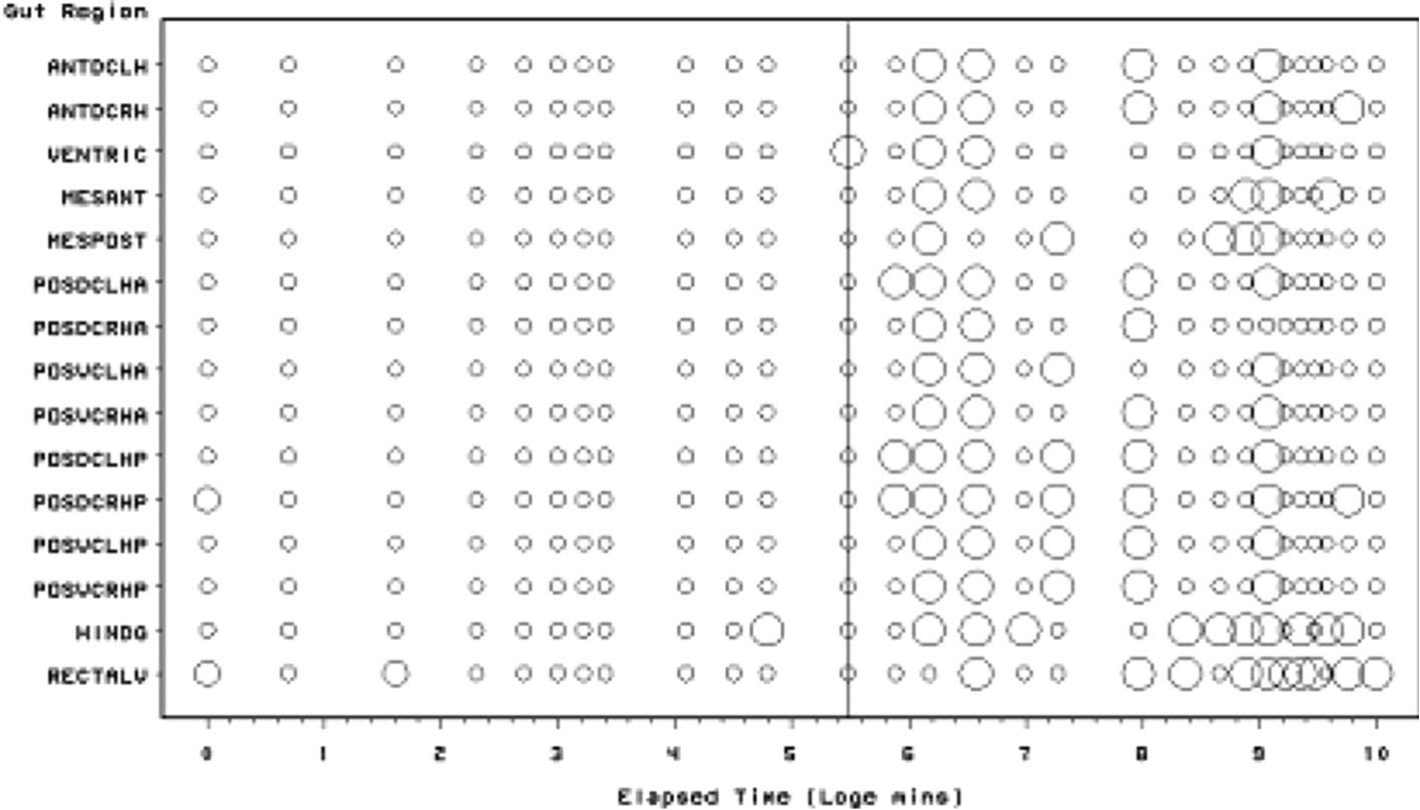
Schematic of *Pergamasus longicornis* lumenal opaque refractive grains in each gut region (ordered anterior to posterior) as: Lots (*large circles*), or, None (*small dots*). *Intermediate size circles* represent presence of some refractive grains or an average over replicates. Time is from the commencement of feeding and is on a natural logarithmic scale. *Grey line* is at the 240 min (=4 h after the commencement of feeding) Bayesian posterior estimate of the change point overall. This marks a marked increase in the presence of lumenal refractive grains and the beginning of apparent final transfer into the hind-gut and rectal vesicle

Scoring of each gut region for the presence of lumenal refractive grains is presented in Fig. 5. The Bayesian posterior estimate of the change point overall is at 240 min (=4 h after the commencement of feeding). This marks a marked increase in the presence of lumenal refractive grains and the beginning of apparent final transfer into the hind-gut and rectal vesicle. Four hours post the commencement of feeding marks the transition from the absence of opaque fine refractive granular lumenal material to its presence. This fine refractive material is present in the lumen of most regions of the gut up to and including 144 h post commencement of feeding i.e. up to when fasting/starvation begins (see Bowman 2014).

**Fig. 6:**
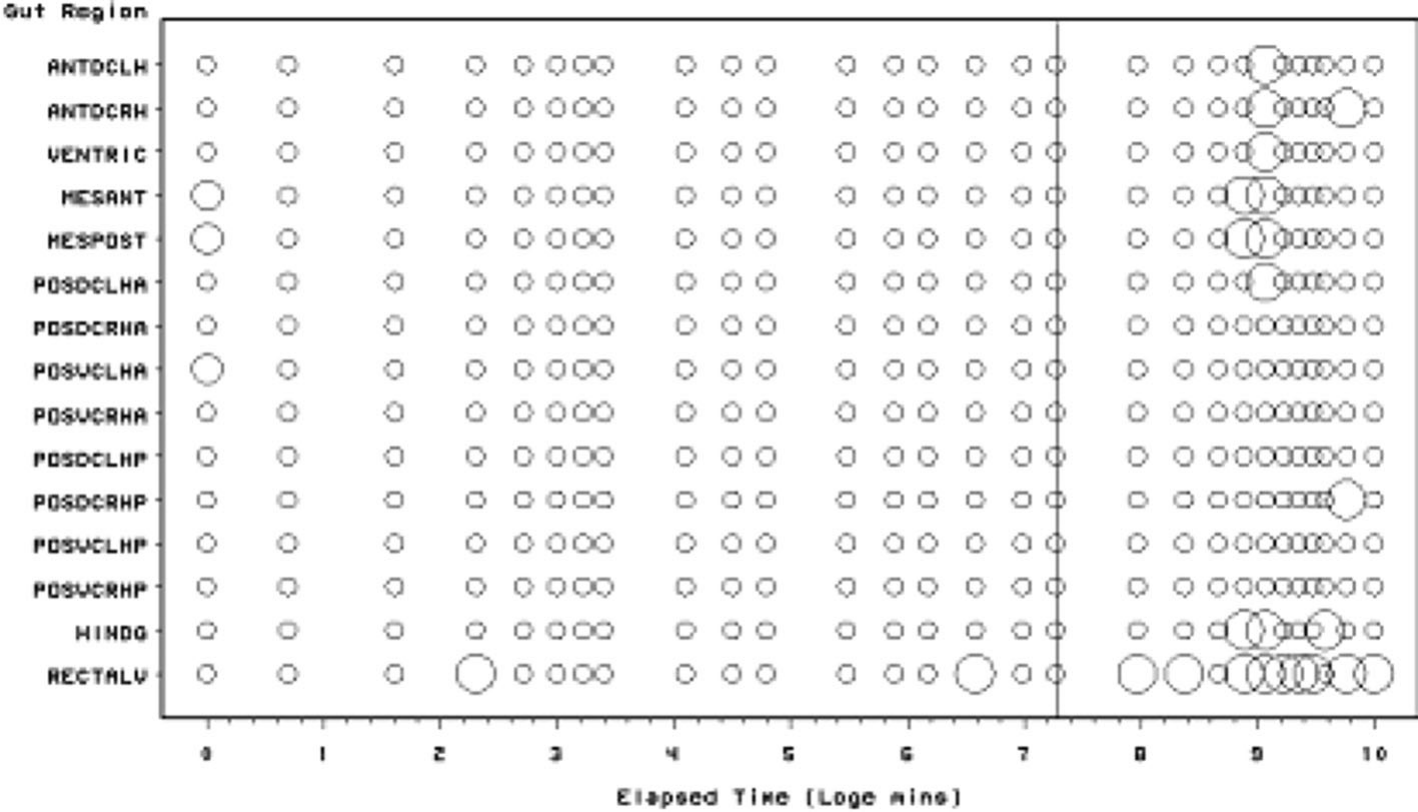
Schematic of *Pergamasus longicornis* lumenal membranous material in each gut region (ordered anterior to posterior) as: Lots (*large circles*), or, None (*small dots*). *Intermediate size circles* represent presence of some membranous material or an average over replicates. Time is from the commencement of feeding and is on a natural logarithmic scale. *Grey line* is at the 1440 min (=24 h after the commencement of feeding) Bayesian posterior estimate of the change point overall. This marks the beginning of an increase in the presence of lumenal membranous material which may represent terminal ‘faeces’ from a meal (current or previous) or perhaps peritrophic membrane precursors? However, note that membranous material is present in the gut lumen of starved mites (time zero)

**Fig. 7:**
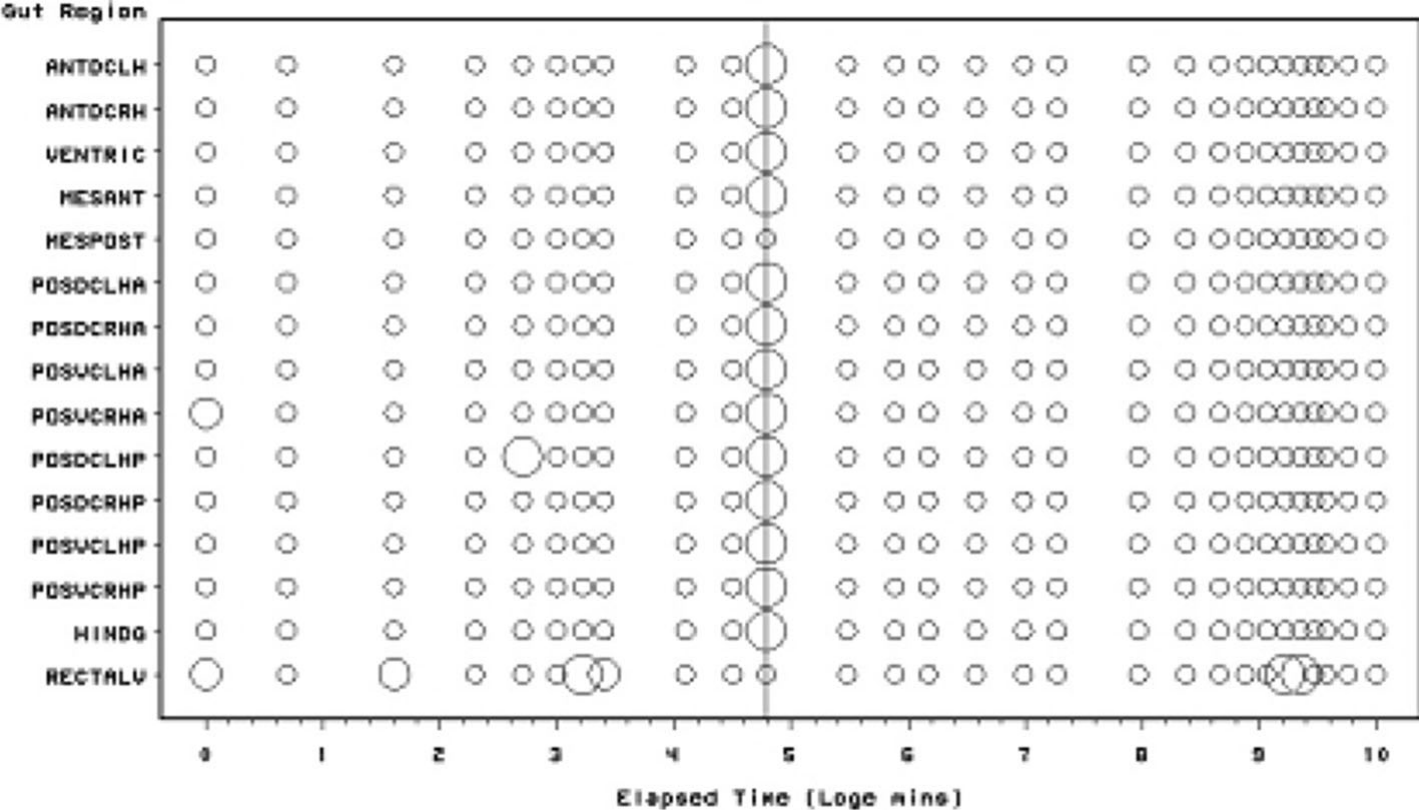
Schematic of *Pergamasus longicornis* lumenal material classed as ‘Other’ in each gut region (ordered anterior to posterior) as: Lots (*large circles*), or, None (*small dots*). *Intermediate size circles* represent presence of some material classed as ‘Other’ or an average over replicates. Time is from the commencement of feeding and is on a natural logarithmic scale. *Grey line* is at the 120 min breakpoint between ingestion dominating and digestion predominating (see Bowman 2014) overall

Scoring of each gut region for the presence of lumenal membranous material is presented in Fig. 6, while scoring of each gut region for the presence of lumenal material classed as ‘Other’ is presented in Fig. 7. Both are sporadic. The Bayesian posterior estimate of the change point overall for membraneous material is at 1440 min (=24 h after the commencement of feeding). This marks the beginning of an increase in the presence of lumenal membranous material which may represent terminal ‘faeces’ from a meal (current or previous) or perhaps peritrophic membrane precursors? However, note that membranous material is present in the gut lumen of starved mites (time zero). Bayesian breakpoint modelling was not carried out on ‘Other’ material.

Note that Bowman (2017) already showed—with the exception of the rectal vesicle—no guanine in the mid- and hind-gut of *P. longicornis* (unlike in oribatids—Smrž 2002).

**Fig. 8:**
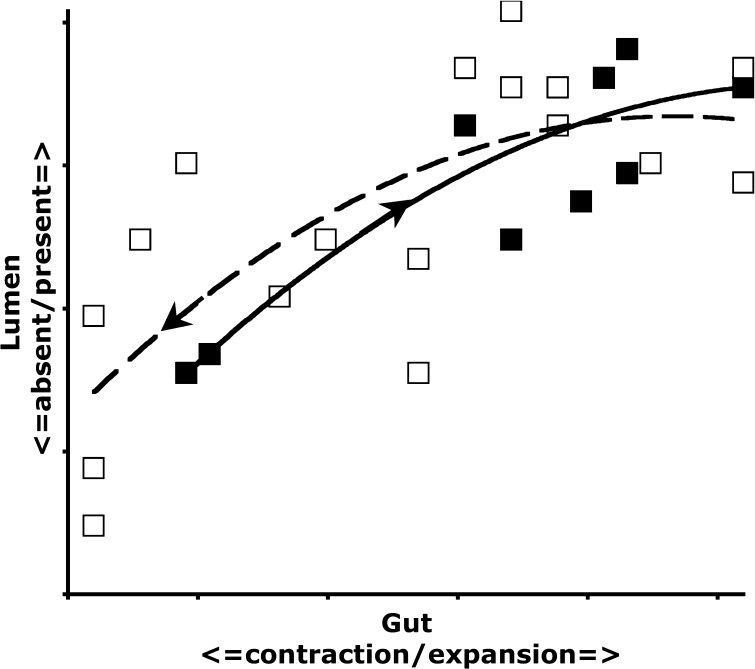
Relationship of lumenal presence with gut expansion from Bowman (2014) showing lack of hysteresis during ingestion/digestion versus digestion/egestion in *Pergamasus longicornis*. *Solid squares* and *solid quadratic trend line*—gut filling-predominating, 0–2 h from the start of feeding on larval dipteran prey. *Open squares* and *dashed quadratic trend line*—gut emptying-predominating, 2$^{+}$–14 days from start of feeding

Figure 8 shows the relationship between the gut lumen scored in this study and the gut expansion/contraction score from Bowman (2014). This shows the lack of hysteresis during ingestion/digestion versus digestion/excretion. A lumen is maintained even when the gut is contracted, however its contents radically change.

**Fig. 9:**
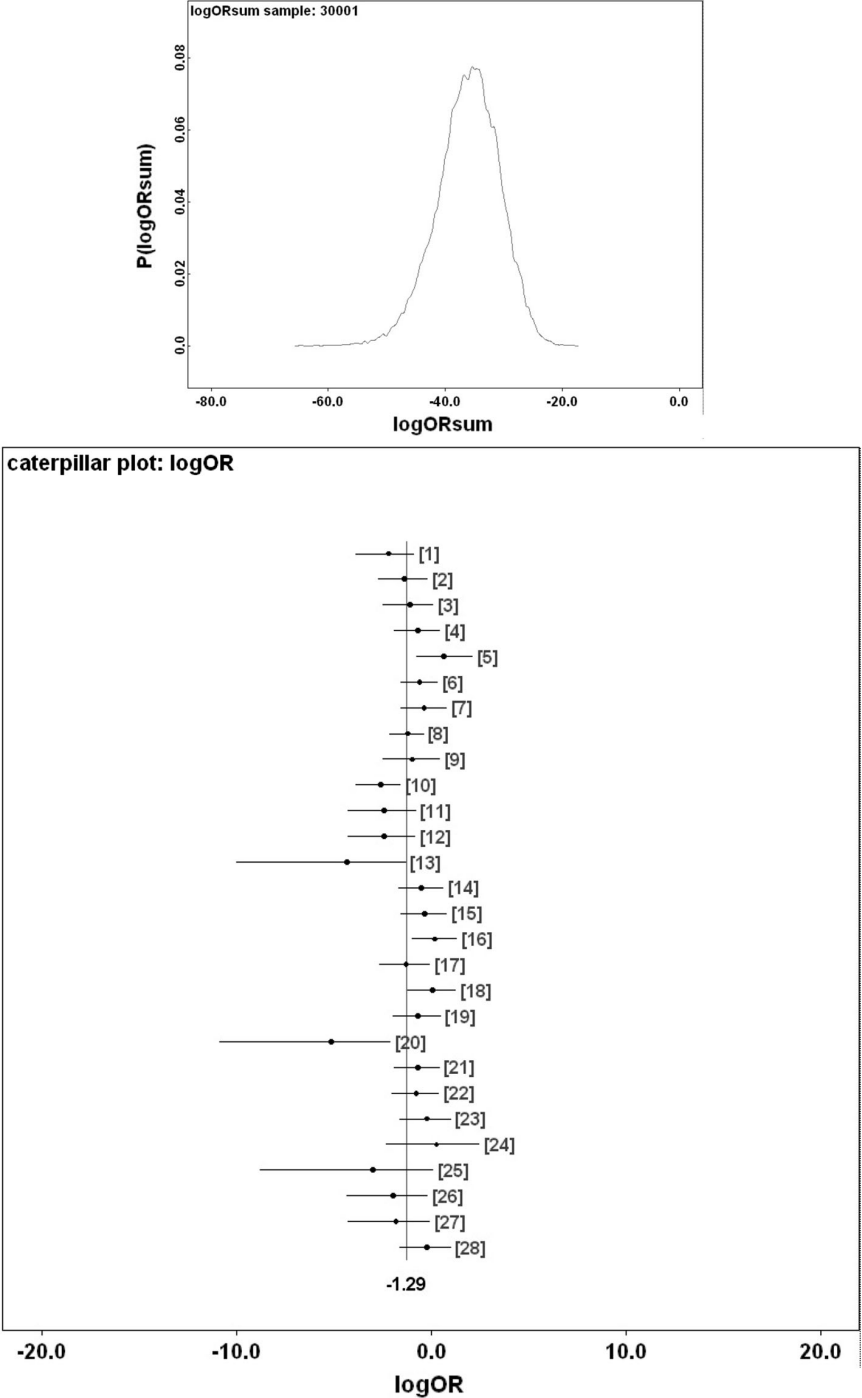
Posterior distribution of log(odds ratio) for the comparison of the lumen presence (see Fig. 3) for anterior versus posterior gut regions (not including the rectal vesicle) in *Pergamasus longicornis*. *Upper* Over all time points. There is strong evidence overall (mean = −36.06, sd = 5.33, $p=0.051$) that anterior regions have a lower occurrence of a lumen than the gut posterior regions. *Lower:* Caterpillar plot for each distinct time point separately (here indexed as logOR[1...28] where [1] = 0 min, [2] = 2 min, [3] = 5 min, [4] = 10 min, [5] = 15 min, [6] = 20 min, [7] = 25 min, [8] = 30 min, [9] = 1 h, [10] = 90 min, [11] = 2 h, [12] = 4 h, [13] = 6 h, [14] = 8 h, [15] = 12 h, [16] = 18 h, [17] = 1 day, [18] = 2 days, [19] = 3 days, [20] = 4 days, [21] = 5 days, [22] = 6 days, [23] = 7 days, [24] = 8 days, [25] = 9 days, [26] = 10 days, [27] = 12 days, [28] = 366 h) after the start of feeding

**Fig. 10:**
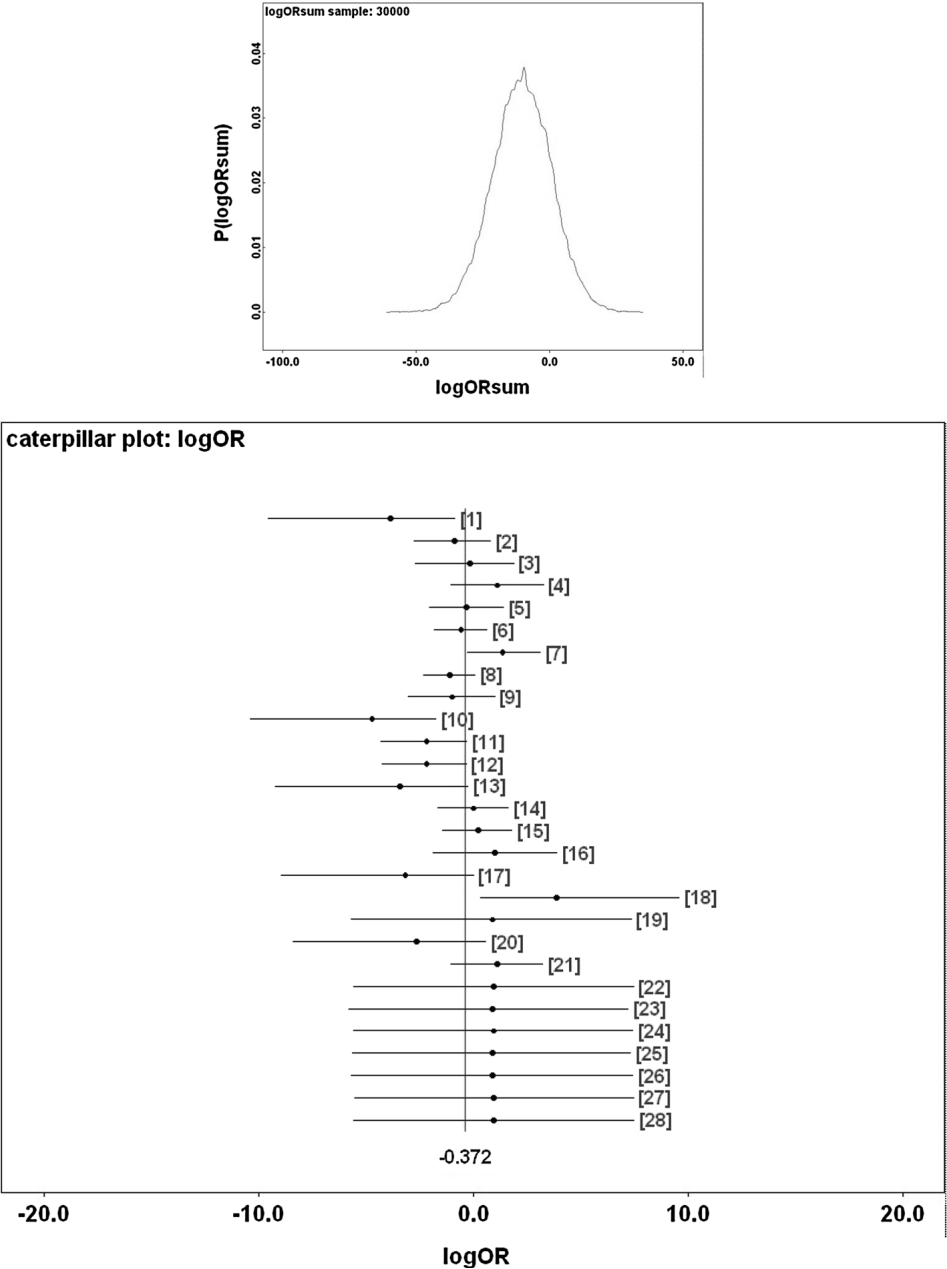
Posterior distribution of log(odds ratio) for the comparison of the granular content in the lumen (see Fig. 3) for anterior versus posterior gut regions (not including the rectal vesicle) in *Pergamasus longicornis*. *Upper* Over all time points. There may be some evidence overall (mean = −10.42, sd = 11.14) that anterior regions have a lower occurrence than the gut posterior regions. *Lower* Caterpillar plot for each distinct time point separately (here indexed as logOR[1...28] see Fig. 9 for times). There appears perhaps to be a higher occurrence posteriorly from 90 min to 4 days after the start of feeding

**Fig. 11:**
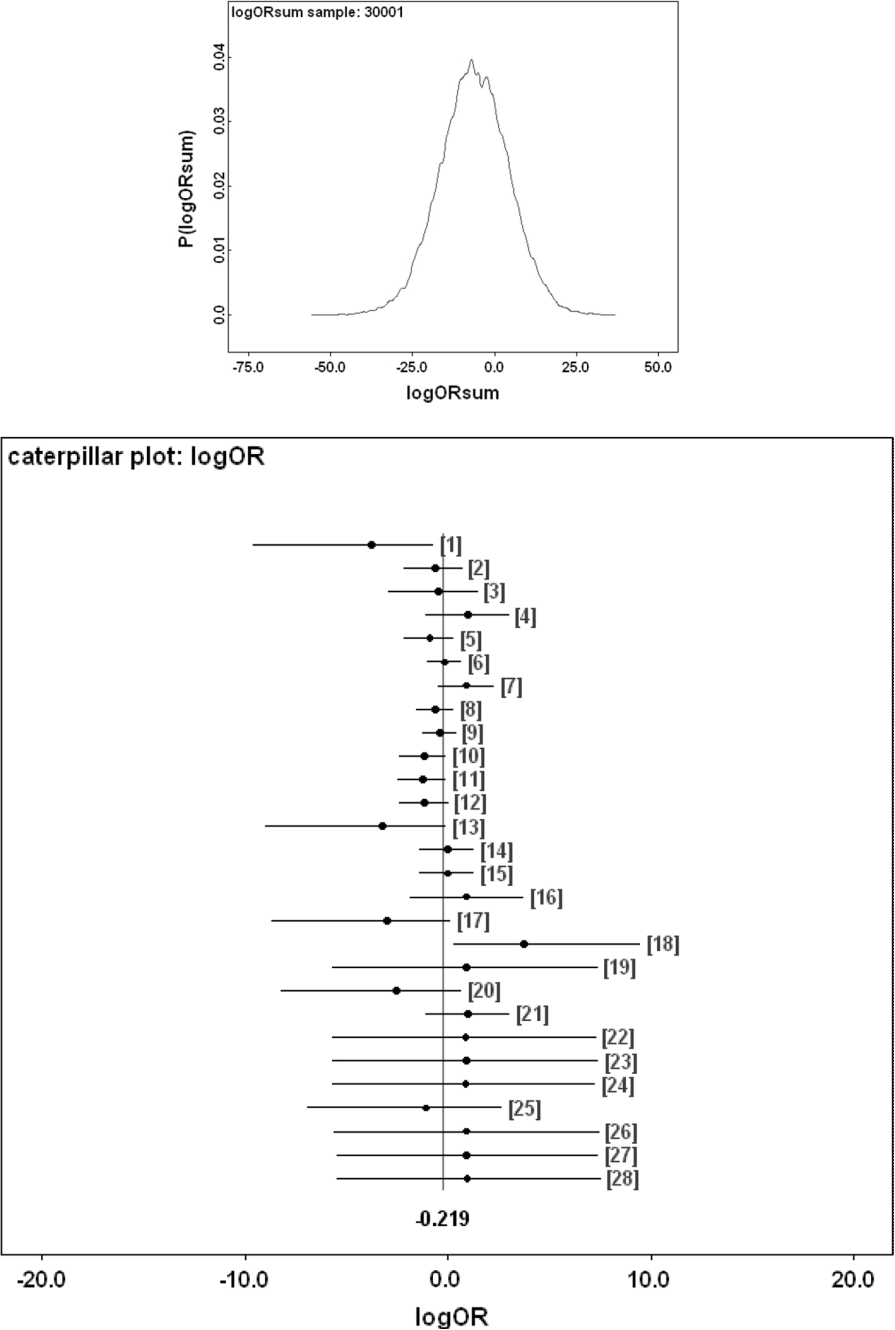
Posterior distribution of log(odds ratio) for the comparison of the globular content in the lumen (see Fig. 4) for anterior versus posterior gut regions (not including the rectal vesicle) in *Pergamasus longicornis*. *Upper* Over all time points. There is little evidence overall if at all (mean = −6.14, sd = 10.52) that anterior regions have a lower occurrence than the gut posterior regions. *Lower* Caterpillar plot for each distinct time point separately (here indexed as logOR[1...28] see Fig. 9  for times)

**Fig. 12:**
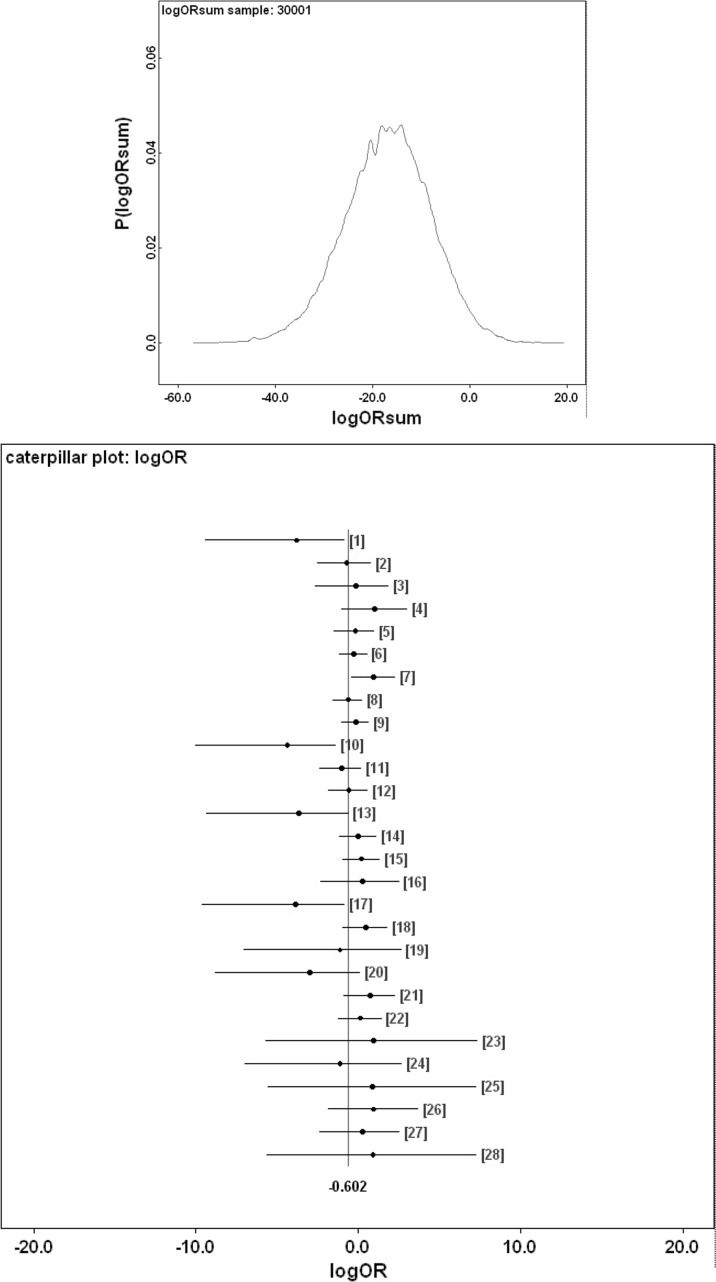
Posterior distribution of log(odds ratio) for the comparison of the opaque refractive grains in the lumen (see Fig. 5) for anterior versus posterior gut regions (not including the rectal vesicle) in *Pergamasus longicornis*. *Upper* Over all time points. There is some evidence overall (mean = −16.86, sd = 8.907) that anterior regions have a lower occurrence than the gut posterior regions (not including the rectal vesicle) This is particularly noticeably for the hind-gut (see Fig. 5). *Lower:* Caterpillar plot for each distinct time point separately (here indexed as logOR[1...28] see Fig. 9 for times)

Figure 9 shows Bayesian posterior distributions for any evidence of an overall difference in the lumen between anterior versus posterior gut regions. There is strong evidence overall (mean = −36.06, sd = 5.33, $p=0.051$) that anterior regions have a lower occurrence of a lumen than the gut posterior regions. Figure 10 shows Bayesian posterior distributions for any evidence of an overall difference in the non-refractive granular lumenal content between anterior versus posterior gut regions. There may be some evidence overall (mean = −10.42, sd = 11.14) that anterior regions have a lower occurrence than the gut posterior regions. There appears perhaps to be a higher occurrence posteriorly from 90 min to 4 days after the start of feeding. Figure 11 shows Bayesian posterior distributions for any evidence of an overall difference in the globular lumenal content in anterior versus posterior gut regions. There is little evidence overall if at all (mean = −6.14, sd = 10.52) that anterior regions have a lower occurrence than the gut posterior regions. Figure 12 shows Bayesian posterior distributions for any evidence of an overall difference in the lumenal refractive grain content between anterior versus posterior gut regions. There is some evidence overall (mean = −16.86, sd = 8.907) that anterior regions have a lower occurrence than the gut posterior regions (not including the rectal vesicle) This is particularly noticeably for the hind-gut (see Fig. 5).

**Table 1:** Estimated digestive status breakpoints and half lives in each simultaneous kinetic phase of processing various lumenal contents during digestion in *Pergamasus longicornis*

Lumen	Time	Switch to	Phase	$\hat{t}_{1/2}$
Presence	5 min	Opening	Appearing	7.2 h$^{*}$
			*Appearing*	
			*(no disappearing)*	
			-*from 0 to 20 min*	4.8 min
			-*from 20 to 90 min*	33 min
			*from 90 min to 8 h*	2.7 h
	6 d	Closing	Disappearing	13 days $$^{**}$$
Granular content	12 h	Disappearing	Input	23 min $^\dag$
			Elimination ph.1 (fast)	6.9 h
			Elimination ph.2 (slow)	4.5 days
			*Elimination overall*	2.7 days $$^{***}$$
			-*Anterior*	5.7 days
			-*Posterior*	2.3 days
Globular content	4 h	Disappearing	Input	36 min $^{\dag \dag }$
			Elimination	1.1 h $^\ddag$
Refractive grains	4 h	Appearing	Input	6.3 h $^\S$
			Elimination	4.5 days

Membraneous and other lumenal contents not modelled. $$^{*}$$ Estimated simultaneously with disappearance over first 8 h. $$^{**}$$ Estimated simultaneously with appearance over 8–366 h. $$^\dag$$ Input half life may be shorter as fitting solely an input phase with no elimination over the first 60 min after the start of feeding through the maximum observed value suggests a half-life of 9 min. $$^{***}$$ Poorly estimated. $$^{\dag \dag }$$ If the input process occurs from time zero (through to 2 h rather than 90 min) then the half life estimate is slightly longer at 52 min. If no simultaneous elimination occurs, a fit over the rise at 25–90 min suggests a longer half-life of 1.1 h. $$^\ddag$$ Estimate based on data up to 8 h—actual half-life may be longer (=2.7 h) if fitted through data including the 12 h time point. $$^\S$$ If no simultaneous elimination occurs, a fit over the rise from 60 to 90 min to 6 h gives a shorter half-life of 2.0–2.3 h

Table 1 shows the estimated change-point and half-lives for the lumen itself and for each type of lumenal content (gut ‘compartment’ of interest). This is discussed in great detail below. No untoward behaviour was detected in any of the sensitivity analysis. Estimates of the location of any change point in processes varied according to the lumenal character measured. With the exception of the appearance and disappearance of the lumen itself, change points for processes were estimated consistently as unimodal. Although not always, the changes in the lumenal contents of the rectal vesicle often appeared roughly 180$^{o}$ out of phase with the lumenal changes in the other gut regions. There was no strong evidence of a contiguous complete peritrophic membrane in the gut as described by Houck (1993) in the ascid *Proctolaelaps regalis*. No structurally recognisable solid prey material was found lumenally anywhere in the pergamasid's gut.

**Fig. 13:**
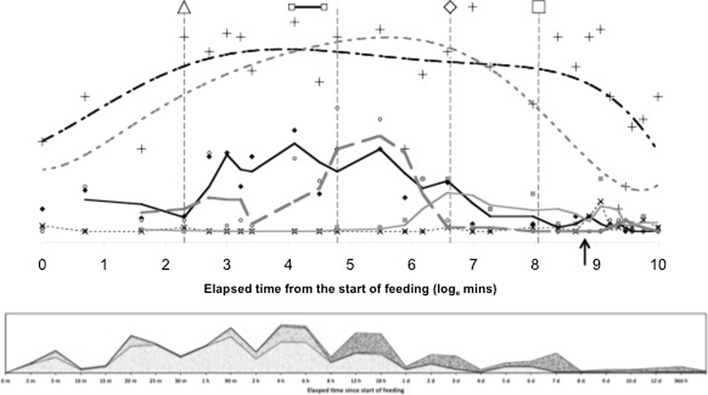
Re-scaled schematic of overall gut lumenal changes during feeding and digestion in *Pergamasus longicornis*. *Upper* Gut expansion/contraction mean score Bowman (2014)—*no symbol*, *upper heavy dashed grey* (6th order polynomial) *trend line*; Lumen presence/absence mean score—crosses and *upper black dashed* smoothed (5th order polynomial) *trend line*—note it does not quite match gut expansion/contraction; *Lower* Granular lumenal contents mean score—*solid diamonds* and *black solid* (second order moving average) *trend line*; Globular lumenal contents mean score—*open circles* and *grey dashed* (third order moving average) *trend line*; Refractive (*dark*) granular lumenal contents mean score—*grey solid squares* and *grey solid* (third order moving average) *trend line*; Membranous lumenal contents mean score—x’s and *light dotted data points line* basally. Time is from the commencement of feeding and is on a natural logarithmic scale. *Vertical central broken line* is at the 120 min breakpoint between ingestion dominating and digestion predominating (see Bowman 2014). Y-axis is arbitrary but does infer relative amounts between plotted points within each line. *Square* indicates worse-case total feeding cycle time 52.5 h based upon Bowman (2014) modelling gut expansion/contraction, thereafter is excretion and defecation. *Triangle* indicates best estimate of initial completion of gut filling time 10 min (see Bowman 2014). Diamond indicates best estimate of time of initial commencement of gut emptying 12.5 h. *Small open squares* joined by *black line* mean feeding times for males (56 min) and females (96 min)—see Bowman (1987). *Black arrow* indicates time of peak Malpighian tubule guanine from Bowman (2017). Note elongate ‘pulse’ of imbibed granular material is ‘chased’ by peak of globular material once feeding is over. Note rise in refractive grains as globular and granular material declines. Note defecation peak of opaque grains after maximum guanine excretion. *Lower* Stacked data for lumenal contents against time point of collection after start of feeding. *Fine grained pale grey* granular lumenal material. *Bubbled grey* globular lumenal material. *Dark coarse grey* lumenal opaque refractive grains

Gut expansion/contraction and lumen are both positively correlated with the appearance of granular material. Membraneous material may be faeces or precursors to a peritrophic membrane (see Discussion). The pulse of imbibed pale granular material is transformed by digestion into globular material, then catabolised and lost. Note the elongate ‘pulse’ of imbibed granular material is ‘chased’ by a peak of globular material once feeding is over (see Fig. 13). Note also the rise in darker refractive grains as globular and granular material declines. These late darker refractive grains are possibly voided from the gut together presumably with any produced faecal material—see defecation peak of opaque grains after maximum guanine excretion (see Bowman 2017).

## Discussion

A first-order input-output model is reasonable to assume for *P. longicornis*—a free-living predator. Experimentally, when phytoseiid predators are transferred from one food source to another, the percentage of the former food in the gut declines exponentially after transfer to a new diet (Sabelis 1981)—91.8% being lost within 1 day, and 99.94% within 3 days. Although labelled as a bolus feeder by Bowman (2014), the initial gulp of prey must be limited by pharyngeal dynamics and oesophageal narrowness in *P. longicornis*. In ticks or haematophagous acarines one might better assume a constant rate input over a fixed time (like an infusion with limitless potential amounts). For pergamasids the input is likely to change over the time of feeding starting high and declining over some time-course form—models of the form used herein are thus more justified. Prey tissues do not disappear immediately over the 56–96 min of feeding but take time to process (see Bowman 1987). A simple exponential model can approximate most non-linear monotonic ascending or declining changes—especially given the other sources of variational noise in this study. Misspecification of the detailed time-course form for deriving summaries is the least of concerns. Larger errors arise from comparing across destructively sampled mites. Just like for the Markov transition probabilities above, half-life and breakpoint estimates are just simply a means to an end (i.e. indicators for biological insight derived from their scale and general consilience) not a precise end in themselves. Indeed half-lives would be expected to vary with the exact type of material consumed—for instance if a mite specialised in more than one type of prey with markedly different composition. However, this is unlikely to be the case for a general arthropod predator like *P. longicornis*.

All ingested prey material appeared fluidised. This supports the view that mesostigmatids employ extracorporeal digestion (like pseudoscorpions—Vachon (1934) and predatory beetles—Evans (1965)) and possibly also use emulsifiers (see Vonk 1962; Collatz and Mommsen 1974) or anticoagulants (known to occur in the saliva and intestines of closely related ticks—Nuttal and Strickland 1908; Pavolvsky and Chodukin 1929; Zhang et al. 2017). Extra-corporeal digestion is already suspected in other mites (see Hamilton et al. 2003).

Unlike during digestion in the acarids *Caloglyphus* and *Tyrophagus* (see Prasse 1967), the gut lumenal contents in *P. longicornis* appear to change reasonably synchronously across every gut region (left and right, anterior and posterior, with the exception of the rectal vesicle which is always lumenate—see Fig. 2). The timing, however, of the changes varies between the different lumenal characterisations (‘compartments’) considered in this study. Physiological and thus ecological insights can be generated by considering each lumen character in turn as below.

### Lumen

The presence of a lumen is broadly correlated with the gut expansion/contraction score used by Bowman (2014) $r^{2}=0.57$ (see Fig. 8). However, it precedes its expansion and post-cedes its contraction somewhat. In particular, a lumen is present despite gut contraction 30$^{+}$ h after the starting of feeding (log$_{e}$ time = 7.5—see Fig. 13). Perhaps a disproportional loss of gut epithelial cells is occurring then? A sensitivity analysis of the Bayesian estimation of the overall change point showed two estimates of high posterior density –5 min and 8640 min (=144 h after the commencement of feeding). These mark first a very rapid appearance in lumenal presence agreeing with the 2–3 min ingestion half-life of Bowman (2014), then a decrease abruptly (marking a return to fasting/starvation—see Bowman 2017) respectively.

Figure 13 displays the results of the gut lumenal status scored in this study averaged over all gut regions against the backdrop of overall gut size changes described by Bowman (2014). Although the appearance and disappearance of the lumen at first look seems homogeneous (see Fig. 8)—broadly tracking the pattern of gut expansion and contraction reported by Bowman (2014)—there is strong evidence overall (mean = −36.06, sd = 5.33, $p=0.051$) that anterior regions have a lower occurrence of a lumen than the gut posterior regions (see Fig. 9). The estimate of 5 min for a switch from no lumen to a lumen being present matches the rapid gut filling (3 min half life) reported previously. A lumen is consistently established by 10 min. However, there are essentially no lumenal contents up to 10 min post the start of feeding showing that the initial ‘gulp’ of dipteran larval prey material is watery and clear. Pumping up of the gut with swallowed air is a possibility but is unlikely. Detectable tangible prey material only appears thereafter during the concentrative phase of coxal droplet production (10–90 min). During this time and up to the end of feeding and the switch from gut filling-predominating to gut emptying-predominating, food contents arrive and increase as the whole gut expands until a lumen is present everywhere. From then up to 52.5 h, the lumen within the expanded gut is replete with imbibed prey material being variously processed (see below).

Kinetically, 3–5 halflives (see Table 1) for the overall appearance of the lumen suggests a zenith of its presence by 21.5 h–1.5 days from the commencement of feeding—that is about half-way through the total feeding cycle of 52.5 h (see Bowman 2014). Ignoring eliminative output (given the order of magnitude difference in kinetics), the early rise in lumenal presence can be seen to have various phases. These are:-

- a very fast initial half-life of approximately 5 min (matching the rapid gut filling half life of 3 min in Bowman 2014);
- an early phase half-life of 33 min spanning the period of initial completion of gut filling at 10 min (Bowman 2014);
- and, a later half-life of some hours spanning the knot point between ingestion dominating and digestion predominating at 2 h (from Bowman 2014).

Three to five halflives of the latter process matches well the 12.5 h for the commencement of initial gut emptying (from Bowman 2014). Whilst it is illuminative to ignore eliminative output early on during prey inhibition, this is an artifice used to just point to the high speed and heterogeneity of the rise in the appearance of a gut lumen. Occam’s razor requires in the first instance to assume physiological processes are contemporaneous (unless there is clear evidence to the contrary). However, it is tempting to ascribe the origin of these three input phases depending upon the actual prey tissues involved in ingestion over time. So through matching, at first perhaps,

- watery/soluble material arrives;
- next, fairly easily extra-corporeally digestible material;
- then, later the breakdown products of the initially tougher residual prey tissues may be ingested.

In this study just one type of non-refractive granular material arrives so immunohistochemical confirmation in follow-up work is needed to confirm or refute this matching. It is congruent with the feeding observations of Bowman (1987).

A slow overall ‘elimination’ half-life for the lumen ‘compartment’ is estimated as 12.7 days (see Table 1). This suggests that even amongst mites starved for at least the same time as in this study (i.e. 2 weeks), a lumen may still be present in the gut—particularly posteriorly (given the log odds ratio result)—for a very long time indeed (i.e. 3–5 half lives is $\equiv$38–63 days). This is certainly confirmed for the posterior parts of the gut in mites at $t=0$ in this study (see Fig. 2)—a lumen appears to be present from the previous meal before the study. The estimate of lumen disappearance beginning at 144 h is much higher than the 52.5 h suggested by Bowman (2014) for digestion to have completed. What might be going on here? Does this larger value bound or mark the end of digestion better? No,—Fig. 13 shows that a lumen persists even when the gut is contracted—however the lumenal contents dramatically change. This apparent conundrum is discussed in the next sections.

### Granular content

Non-refractive granular material is the first tangible prey material to appear in the gut system (see Fig. 3). There is no clear evidence that the anterior parts of the midgut behave like a ‘first-in’ (FI) system as claimed by Bowman (2014). A sensitivity analysis of the Bayesian estimation of the overall change point indicates that the change point for the granular content ‘compartment’ is at 720 min ($\equiv$12 h after the commencement of feeding). This marks a noticeable collapse in the presence of lumenal granular material—all ingested prey material has essentially gone after this point from all mid- and hind-gut regions. For this granular lumen content, there may be some evidence overall (mean = −10.42, sd = 11.14) that anterior regions have a lower occurrence than the gut posterior regions (see Fig. 10). There appears perhaps to be a higher occurrence posteriorly from 90 min to 4 days after the start of feeding. Given that Bowman (2014) shows that the switch from gut filling-predominating to gut emptying-predominating is at 2 h after the start of feeding and Fig. 13 shows granular material to be present on average from about 15 min to 1 day or so, this suggests that while the whole mite gut fills synchronously with this prey material its disappearance may take longer from the posterior gut regions. This disparity could be evidence that at least in part its disappearance is due to intracellular processing, as extracellular processing in the lumen would be expected to be uniform throughout the interconnected lumen of the whole gut system. Of course, the larger amount of foodstuff in the larger posterior parts of the mid-gut could take longer to process if saturable Michaelis-Menton kinetics applied to its digestion. Further biochemical tracking work is needed.

Pergamasid prey must be liquidised before ingestion by some extra-corporeal digestion as larval dipteran prey are completely consumed bar their cuticle (Bowman 1987) just like phytoseiid mites totally devour the insides of tetranychids (see Flechtmann and McMurtry (1992). For sure, just like host erythrocytes in tick guts (Sojka 2015), prey cells are not just lysed but must be completely digested. Whether collagenase, proteases and DNAse enzymes are injected in with a venom as in snakes and spiders (Kaiser and Raab 1967; Stahnke and Johnson 1967) or with a local toxin as in ixodids (Nuttal and Strickland 1908) and scorpions (Zlotkin et al. 1971), or introduced by regurgitation, it is not clear. Genome sequencing has confirmed neurotoxin like and sphingomyelinase genes in the predatory phytoseiid *Metaseiulus occidentalis* (Hoy et al. 2016). It is tempting to suggest that salivary products injected via the long salivary styli found on both sides of the mesostigmatid gnathosoma (see Gorirossi 1955; Bohley and Seglen 1989) are the cause. Flechtmann and McMurtry (1992) reports evidence for (salivary based?) proteolytic enzymes injected into the prey in a variety of phytoseiid mites with at least some pre-oral digestion. Rather than salivary or gut produced, Nuttal and Strickland (1908) reports that argasid coxal fluid has an anticoagulant effect, so whether pergamasids also introduce such into their prey through re-circulation of their coxal fluids (Bowman 2014) via the gnathosomal groove and tritosternum (Wernz and Krantz 1976) remains to be seen.

The non-refractive ingested granular material appears in the gut lumen with a very short half-life of appearance (23 min or less—see Table 1). Much as the green, red or dark particulate material observed in the gut of phytoseiids (Flechtmann and McMurtry 1992), this must represent partly or unbroken down prey material as well as perhaps opaque precipitated-out products from the initial imbibition of clear fluid. As the granular material *P. longicornis* was homogeneous on microscopic inspection yet the larval dipteran prey tissues are macroscopically heterogeneous, it suggests a screening/straining mechanism in the mite’s oral apparatus (perhaps by the pre-oral channel spicules under the labrum—see Evans and Loots 1975; Flechtmann et al. 1994) is used. Note that this half-life is several times longer than the 3–8 min half-life for initial gut expansion (Bowman 2014) showing that ingested (clear) prey fluid must drive the initial size change in the midgut. The predominance of granular material from 20 min or so after feeding through 2 h (and beyond) i.e. during the concentrative period of coxal droplet production (Bowman 2014) suggests that precipitation from the initially ingested clear fluid as well as granular material ingestion in its own right must also be a source. Similar support arises from noting the fact that the ‘middle’ kinetic phase above for the lumen changes is consilient with the time to complete initial gut filling, yet there being only a single input half-life phase for the granular prey material (Table 1).

**Fig. 14:**
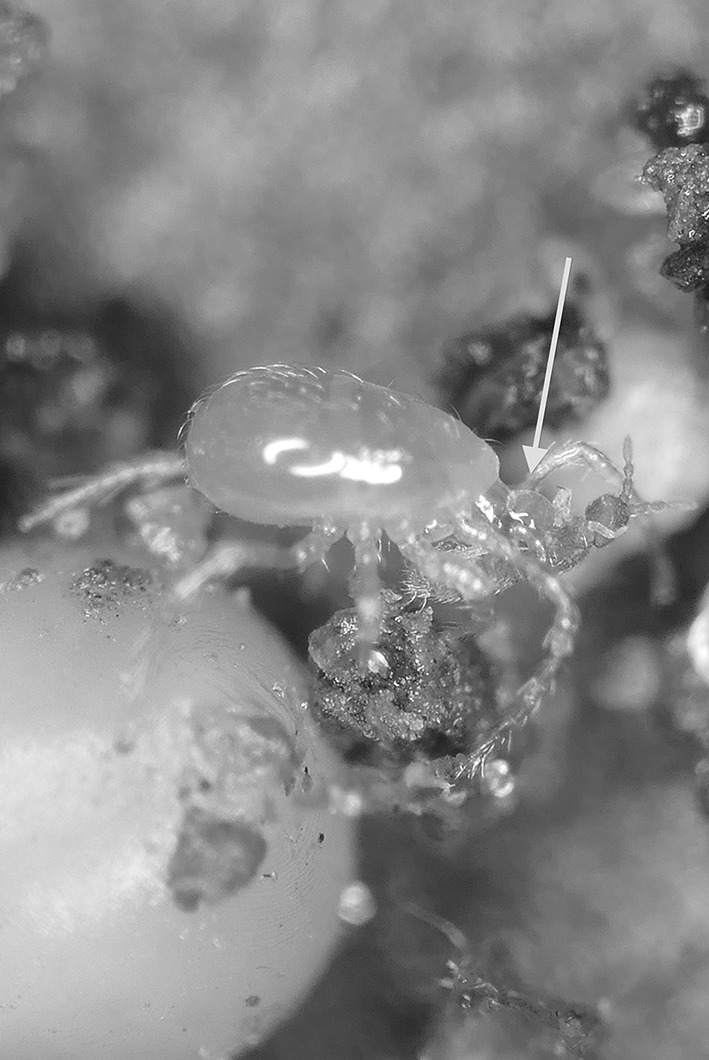
Soil parasitid early on in time of feeding on a springtail. *White arrow* shows clear fluid droplet from ruptured prey over the mite’s gnathosomal area. Once imbibed, this liquid from the haemocoel of the prey is the cause of the initial midgut expansion and early appearance of the gut lumen. Note beginning of a coxal droplet (Bowman 2014) appearing ventrally between coxa I and II. From a colour photograph ©Lennart Bendixen with permission

Given all the above, and by virtue of its large AUC (area under the time curve), I infer that this granular material represents the main tranche of ingested prey tissues—the primary input. That is, the initial fluid handling on prey body rupture—see Fig. 14. This could be confirmed immunologically or spectroscopically in future work (see Zoltowski et al. 1978). Given the high speed of intake (one fly larva as large as the pergamasid consumed in about 1 h) it suggests it is an energetically rich food source (unlike that in phytophagous oribatids—see Hubert and Šustr 2001). Turning then to hypotheses ‘(i)’, ‘(ii)’ and ‘(iii)’. Three to five absorption (i.e. lumenal input of this material) half-lives (i.e. to 88–97% completion) of 69–114 min agrees well with the 56–96 min estimate of average feeding time given by Bowman (1987). Moreover, the stripping process indicates a knot or switch point from absorption predominating to elimination predominating nicely around 60 min from the start of feeding of larval dipteran prey. If no elimination occurred in the first feeding phase swelling the gut out (see Bowman 2014) then the estimated input half-life could be much faster (see Table 1) and would better match the 3–8 min gut expansion half-life (see Bowman 2014). However, Occam’s razor applies—this suggestion is possible but physiologically unlikely as it would need elimination to be specially switched on sometime after feeding commenced. If this occured (i.e. ‘hypothesis(i)’ or ‘hypothesis(ii)’ is true) micro-histological inspection of gut cells would be critical in confirming that digestion within the idiosoma (as opposed to extra-corporeal) does not start until later. In fact, the apparent close matching of the magnitude of the half-lives (see $\dag$ in Table 1) would support ‘hypothesis(ii)’ more than ‘hypothesis(i)’. Given that the peak of granular material is at 60 min, and the end of feeding was estimated by Bowman (1987) as 56–96 min then it is possible that ‘hypothesis(iii)’ may be true. However, if follow-up histological examination of the gut cells before this point shows any evidence of intracellular digestion then ‘hypothesis(iii)’ will be refuted.

**Table 2:** Comparative approximate half-lives for disappearance of food material during acarine digestion (sample based upon a non-comprehensive survey of the literature)

Species	Material	Measure	$\hat{t}_{1/2}$	Data source	Scope
ACARINA					
*Argas persicus* male	Blood	mg Hb/mg initial wt	7.5 d	Fig. 13 Tatchell (1964)	3–12 days
female	Blood	mg Hb/mg initial wt	2.9 d	Fig. 13 Tatchell (1964)	3–12 days
male	Blood	mg Hb/mg initial wt	3.8 d	Fig. 14 Tatchell (1964)	1–14 days
female	Blood	mg Hb/mg initial wt	4.2 d	Fig. 14 Tatchell (1964)	1–14 days
*Dermacentor variabilis* female	Rabbit	Lumen protein mg/ml	8.0 d	Fig. 24a Tarnowski and Coons (1989)	0–33 days
		Gut cell protein mg/ml$^\dag$	4.8 d	Fig. 24a Tarnowski and Coons (1989)	1–7 days
		Gut cell protein mg/ml	5.8 d	Fig. 24a Tarnowski and Coons (1989)	7–33 days
		Lumen Haemoglobin mg/ml	11.0 d	Fig. 24b Tarnowski and Coons (1989)	1–43 days
		Gut cell Haemoglobin mg/ml	9.9 d	Fig. 24b Tarnowski and Coons (1989)	1–43 days
		Lumen Haematin mg/ml	*‘flat’*	Fig. 24c Tarnowski and Coons (1989)	1–43 days
		Gut cell Haematin mg/ml$^\dag$	12.0 d	Fig. 24c Tarnowski and Coons (1989)	1–22 days
		Gut cell Haematin mg/ml	12.3 d	Fig. 24c Tarnowski and Coons (1989)	22–43 days
*Haemaphysalis longicornis * nymph	Rabbit	Protein $\upmu$g/midgut	2.4 d	Fig. 1 Koh et al. (1990)	0–9 days
		Haemoglobin $\upmu$g/midgut	2.8 d	Fig. 2 Koh et al. (1990)	0–9 days
		Albumin $\upmu$g/midgut	9.0 d	Fig. 2 Koh et al. (1990)	0–9 days
		Haematin $\upmu$g/midgut$^\dag$	9.7 d	Fig. 3 Koh et al. (1990)	0–11 days
		Haematin $\upmu$g/midgut	1.1 d	Fig. 3 Koh et al. (1990)	11–17 days
*Ixodes scapularis* nymph	Mouse	Albumin coverage	65.6 d	Table 1 Laskay et al. (2013)	1–120 days
		Albumin peptides $^{\ddag }$	58.9 d	Table 1 Laskay et al. (2013)	1–120 days
		Transferrin coverage	73.3 d	Table 1 Laskay et al. (2013)	1–211 days$^*$
		Transferrin peptides$$^\ddag$$	74.8 d	Table 1 Laskay et al. (2013)	1–211 days$$^{**}$$
		Haemoglobin $\alpha$ coverage	86.3 d	Table 1 Laskay et al. (2013)	1–211 days
		Hb $\alpha$ peptides$^\ddag$	107.8 d	Table 1 Laskay et al. (2013)	1–211 days
		Haemoglobin $\beta$ coverage	107.9 d	Table 1 Laskay et al. (2013)	1–309 days
		Hb $\beta$ peptides$^{\ddag }$	110.5 d	Table 1 Laskay et al. (2013)	1–309 days
*Ornithodoros moubata*	Fowl blood	Occurrence	86.1 d	Table II Weitz and Buxton (1953)	5–150 days

Re-analysis of published figures using simple log-linear regression. $$^\dag$$ appearance. $$^\ddag$$ relative number of unique peptides. $$^{*}$$ $$\hat{t}_{1/2}$$ reduces to 8.0 days for scope of 1–15 days. $$^{**}$$ $$\hat{t}_{1/2}$$ reduces to 7.6 days for scope of 1–15 days. For further references see Obenchain and Galun (2013)

By its persistence through to 1 day or so, the primary tranche of granular food clearly takes the poikilotherm *P. longicornis* a long time to fully process. Slow food breakdown may be typical of mites much as the extended erythrocyte breakdown times found in ticks (see Kirch et al. 1991). A slow elimination phase and a fast elimination phase for the granular material is indicated kinetically (Table 1), i.e. elimination itself is also itself stiff—an advantage for an intermittent feeding predator. Some food is instantly handled, other is at leisure. Whether this reduced digestion is related to mating as in ticks (Tarnowski and Coons 1989) remains to be investigated. One might conclude from this that granular prey material is extracellularly converted in the lumen slowly ($t_{1/2}=4.5$ days, despite acarine gut lumens being thought to be free of digestive enzymes—Coons et al. 1986). It certainly is absorbed into gut cells quickly ($t_{1/2}=6.9$ h). More likely is that this slow rate is processing by slow intracellular digestion (as in other arachnids—see Phillipson 1961). The latter speedy absorptive elimination from the lumen is in broad agreement with estimates of an initial gut clearance half-life of 6.1 h in caeculid mites (Crossley and Merchant 1971) and the 6.7 h half-life calculated from the relative rate of gut emptying from Dicke et al. (1988) used for discussing the phytoseiid *Amblyseius potentillae* in Dicke et al. (1990) (i.e. $\hat{t}_{1/2}=24*\frac{ln(2)}{2.5}$ h). It also nicely spans the 2–8.5 h range of half-life estimates given in Bowman (2014) for elimination based upon gut expansion/contraction alone. Looking at elimination overall (and in particular that posteriorly) the half-life of 2.3–2.7 days matches nicely with the order of magnitude for those found early on in the feeding process of ticks (see Table 2). The second phase slow elimination half-life of 4.5 days in *P. longicornis* is consilient with the tick range (1.1–12.3 days) through to quite long post-feeding times too. A common process may thus be present between these acarine species. This could be expected if overall elimination was dependent upon the same intracellular epithelial mechanism (note that only insects are *known* to digest extracellularly). Whatever that microhistological follow-up might show, neither half-life is consilient with the half-life of pollen being less than an hour in the predatory phytoseiid *Amblyseius swirskii* (Schuldiner-Harpaz et al. 2016). Perhaps imbibed pollen material is much more assimilable directly into mite cells than macerated prey tissues?

**Fig. 15:**
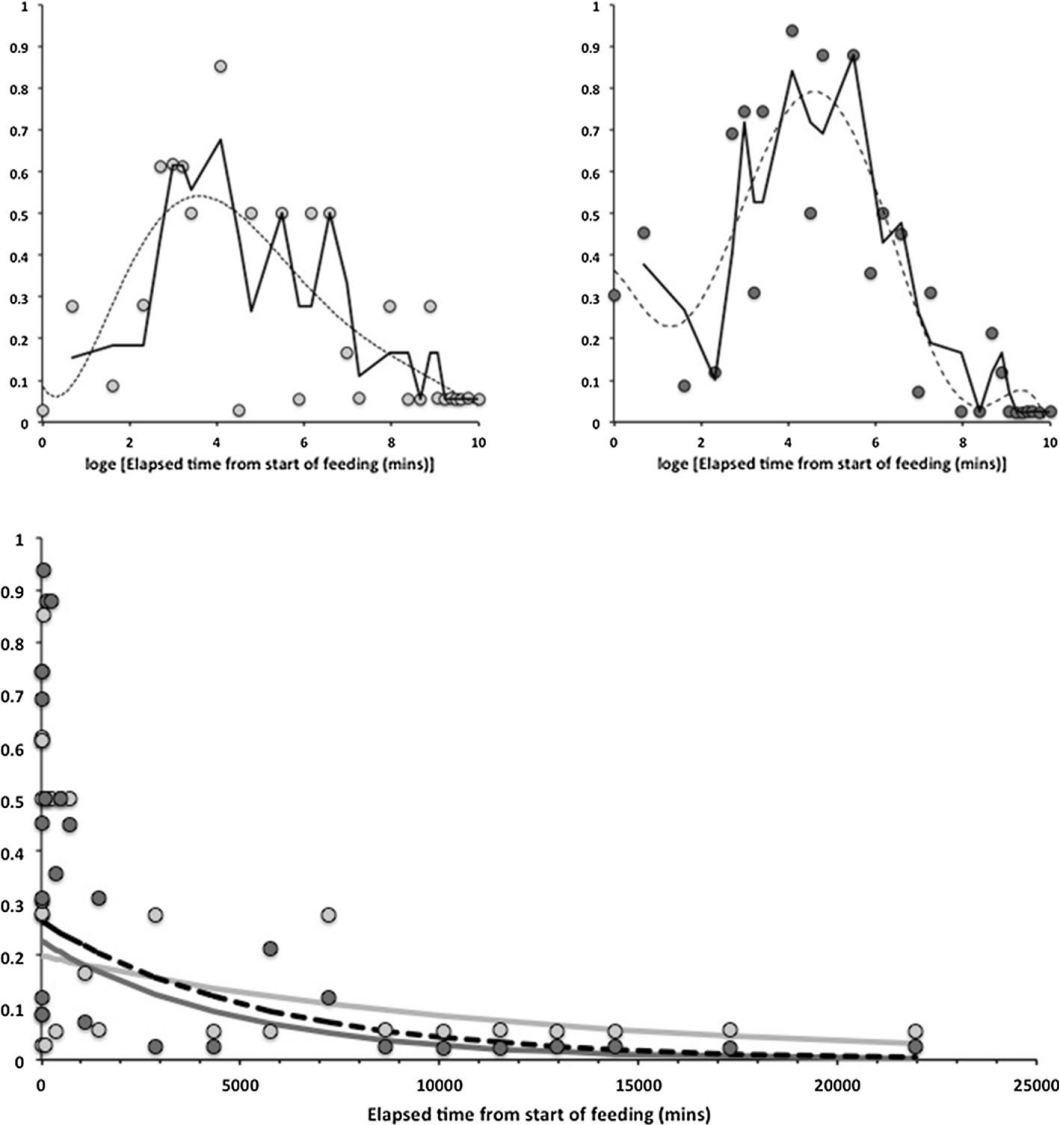
Granular ingested prey material in the gut lumen of *Pergamasus longicornis* assuming single overall phase of elimination—see Table 1. *Vertical axis* equals probability. *Upper*: Spots are mean estimate of occurrence on log time scale. *Solid line* second order moving average. *Dotted line* sixth order polynomial trend. Note similar input profiles but different output forms. *Left* *Pale grey* for anterior gut regions—slower eliminative fall. *Right* *Dark grey* for posterior gut regions—faster eliminative fall. *Lower* Combined data on arithmetic time scale after commencement of feeding. *Symbols* and *colours* as before plus fitted elimination values for that region. *Black dashed line* overall fitted elimination ($\hat{t}_{1/2}$ = 2.7 days). Note poor fits immediately after initial ingestive rise indicating presence of an early rapid conversion phase (see Table 1)

Consider the results of the log odds ratio test in Fig. 9. Is there other matching evidence of slightly different kinetics between the anterior and posterior regions of the midgut? Bowman (2014) claimed the posterior regions to have the expansion/contraction characteristics of a last-in-first-out (LIFO) temporary depot. However, the posterior gut in total is (always) bigger than the anterior gut in *P. longicornis* and therefore teleologically presumably the last to empty i.e. it could be a last-in-last-out (LILO) persistent store of prey food. Which is it? Figure 15 confirms this greater extent of granular material posteriorly (compare peaks between *Left* and *Right* graphs), but their input is similar. For sure then, more prey material does go in posteriorly to the lumen of the swelling gut i.e. it is LI by virtue of its volume—even though it fills at the same rate as that anteriorly. So, the real difference between the midgut regions can only be in their lumenal content output kinetics (i.e. the opposite of ‘hypothesis (iv)’ is confirmed). Too much noise in the data precludes very good estimates for fitting two eliminative phases to each of the regions separately, but Table 1 and the *Lower* graph of Fig. 15 confirms that overall the anterior part of the midgut may have slower output kinetics. Thus for the same amount of material in it as in a posterior part, the anterior part would take the longest to empty (making the posterior region physiologically FO as a corollary). However, the posterior regions are so large that effectively with respect to prey material they are observed as LO (so the posterior midgut regions *are* LILO overall). Bowman (2014) deemed the anterior part to have in part FIFO (first-in-first-out) size characteristics, is this supported? With respect to prey material, the ‘FO’ part of this is probably still true (see Fig. 3)—it is just that its diminution over time is comparatively slow and it looks LO. Given that the peak of lumenal granular material in the anterior parts is slightly earlier than that of the posterior parts (so it is FI—see Fig. 15 *Upper*) and such material persists across all the midgut for a long time—then given the smaller volume, the FIFO designation of the anterior gut with respect to prey contents is to be expected. Perhaps this persistence is a sub-fraction of prey material possibly highly resistant to digestion (see discussion on globular material below)? Further work examining the micro-histology of gut cell changes over time should confirm if the posterior gut does indeed follow a LILO rather than a LIFO process and if there is any confirmation of this mild difference in rate behaviour anteriorly too.

How intracellular ‘waste’ material, which might be produced, is moved into the gut lumen (if at all) afterwards is not clear. Inspection of gut cell histology may clarify this. Perhaps cells degenerate or slough off as in ticks (Tarnowski and Coons 1989)? Differential cell epithelial loss posteriorly would shrink the gut quickly (i.e it would be LIFO), even if lumenal prey material depots within it were taking the longest to clear (i.e. they were LILO). Early filling anteriorly would quickly swell the gut there and given its relatively small size it would appear to quickly diminish on any cell degeneration (i.e. it would look FIFO with respect to size) even when there is an extended clearance of small amounts of lumenal material in it (i.e. and thus appear to be FILO). The switching breakpoint of granular prey material contents at the 12 h post-feeding mark (Fig. 3) is around the time (18 h) that the ‘stripping’ estimation process suggests that the fast early elimination phase is overtaken by the slow eliminative phase dominating. Note that the 3–5 ‘fast elimination’ half-lives equaling 21–34 h also broadly matches this. This time thus may represent the point where intracellular digestion of the primary prey material is coming to a conclusion. Examining gut epithelial cell dynamics could confirm or refute this. However, there is no clear evidence of very large half-lives as in extended observations of some ticks (see Table 2). Three to five ‘slow elimination’ (lumenal disappearance) half-lives (i.e. to 88–97% completion) of 14–23 days exceeds somewhat the estimate of a complete cycle of feeding, digestion, egestion and excretion of 9 days by Bowman (2017). ‘Hypothesis (viii)’ is thus refuted. Whilst hunger/starvation may commence around 10 days, prey food may linger at very low levels in the gut (and perhaps inside the gut epithelial cells) for long periods of starvation in this mite—an undoubted advantage for a predator faced with rare or hard-to-catch prey. Examination of gut cells in starved mites should confirm or refute this.

Given the weight of evidence above, the sporadic nature of some isolated scoring of granular material late on in the rectal vesicle (Fig. 3) suggests that this is either an artefact of scoring or perhaps the final discharge of a small amount of highly indigestible (enzyme resistant) imbibed prey material along with faeces.

### Globular content

Compared to the input of granular material—a degree of hysteresis is indicated overall for the globular lumenal material (compare the two peaks in Fig. 13). I suggest that this initial pattern represents:-

- first the early sporadic extra-corporeal digestion of the prey in situ;
- then, the later consistent extracellular breakdown products of at least part of the earlier ingested prey fluids—perhaps via the formation of free lipids?

For sure, lumenal extracellular digestion of erythrocytes occurs in *Ixodes* spp. ticks as evidenced by their heamolysis in situ (Grigor’eva 2003). Four hours post the commencement of feeding in *P. longicornis* marks the abrupt synchronous transition from the presence of this globular lumenal material to its complete absence (see Fig. 4) with an elimination half-life of 1.1 h (Table 1). This is after the time of the swap from ingestion predominating to digestion predominating (at 2 h—Bowman 2014) but before the breakpoint for lumenal granular material (see Fig. 3). A sensitivity analysis of the Bayesian estimation of the change point for globular lumenal material indicates that the change point is at 240 min ($\equiv$4 h after the commencement of feeding). This marks a noticeable decline in the presence of lumenal globular material. Compared to the levels of granular material declining at this point, proteresis is indicated. In other words, the globular prey material apparently is either rapidly absorbed, or rapidly catabolised *in situ* to something else (see Fig. 13). In part, this is evidence to confirm ‘hypothesis (v)’ although a biochemical link to the sharp rise in guanine production would need confirmation. It would seem that catabolism may be poorly assayed by histological measures, so a different investigative approach is needed to answer this question ‘(v)’ definitively in followup work.

It is tempting to conclude that the lag in the main initial rise of globular material around 1 h post commencement of feeding compared to the initial rise in lumenal granular material, together with the persistence of granular material after the disappearance of lumenal globular material, all indicates the drawn-out conversion of only a certain fraction of the lumenal granular material into globular material over time in *P. longicornis*. Also the apparent ‘sharpening’ (around the 30 min–2 h period) rather than an expected attenuation of the granular input pulse (under linear system kinetics), suggests that this rise in globular material could be by the rapid action of a cascade of say extra-cellular lipases on prey lipoproteins (resulting in the leaving a residue of proteinaceous material for slower digestion later in the gut). This therefore, again partly confirms ‘hypothesis (v)’. The half-life of this sharp rise (fitting a kinetic model from time zero allowing for simultaneous elimination), is between 36 and 52 min (see Table 1). For sure, a fast half-life would be exactly expected from an enzymic process in solution—within a lysosome for instance endopeptidase proteolysis has a half-life as low as 8 min (Bohley and Seglen 1992). Perhaps the acidic nature of the gut lumen in mites (Erban and Hubert 2010) facilitates this. If estimated from the beginning of the rapid increase through the peak maximum—given no elimination occurring—the half-life of the appearances of globular lumenal material is a little longer at 1.1 h. More histological samples are needed over this critical period to determine this better—with the backup of soluble biochemical assays of gut extracts or immunoelectrophoretic dissection (see Meng et al. 1980) too. Either way, complete input (3–5 half-lives) for globular material would be achieved by 3.0–5.6 h, markedly shorter than the presence of granular material (Fig. 13). This again supports the idea of rapid degradation of just a labile fraction within the ingested prey material to leave a possibly more resistant moderately persistent residue. Differential assays of the granular material showing it changes biochemically over time, even though it retains the same light microscopic form, would help in future work.

For the globular lumen content, there is little evidence overall if at all (mean = -6.14, sd = 10.52) that anterior regions have a lower occurrence than the gut posterior regions (see Fig. 11). Unlike with the granular lumenal material, this suggests that the whole mite mid- and hind-gut fills and empties synchronously with this globular material. This suggest that (extracorporeal and) extracellular digestion may be at work as the location over the gut epithelium does not seem to matter. Intracellular processing does not appear to be indicated. Inspection of gut cell histology may confirm or refute this. Given the weight of evidence above, the sporadic occurrence of globular material late on in the rectal vesicle (Fig. 4) may represent evidence for a separate degenerative process (likewise for any rectal vesicle granular material seen in Fig. 3).

### Refractive grains

These tiny dark grains are very distinct from the large excretory crystals found in the Malpighian tubules by Bowman (2017). Confirming if they are or are not made of such guanine could be done by methyl green-pyronin in future work (see Romeis 1948). A sensitivity analysis of the Bayesian estimation of the overall change point for lumenal refractive grains indicates that it is at 240 min ($\equiv$4 h after the commencement of feeding—just as globular material goes into sharp decline). This point marks a marked increase in the presence of lumenal refractive grains and the beginning of an apparent final transfer of material into the hind-gut and onto rectal vesicle storage. A physiological switch in role in the epithelium may be occurring? Micro-histological examination of gut epithelial cells would help in any future work.

For refractive grains in the gut lumen, there is some evidence overall (mean = −16.86, sd = 8.907) that anterior regions have a lower occurrence than the gut posterior regions (not including the rectal vesicle—see Fig. 12). This is particularly noticeable for the hind-gut (see Fig. 5). This suggests that while the whole mite gut fills synchronously with this refractive granular material its disappearance does take longer from the posterior gut regions. This could be explained as an attenuated matching ‘echo’, in the posterior gut regions, of the eliminative kinetics of the original fine granular prey material within the different sizes of the gut regions (see discussion section above)—in line with the proposed system model of Bowman (2017). This idea is obliquely supported by the fact that in at least one tick, haematin handling by the gut shows ‘flip-flop’ kinetics (i.e. rate of disappearance similar to rate of appearance—see Table 2). Consilience of half-lives of the appearance (from a previous compartment) with the half-lives of disappearance from the current department in a physiological model suggests, (given instantaneous partitional equilibrium) clear coupling at least, if not actual causative conversions.

Importantly, refractive granular material persists in quantity in the hind gut and in major amounts within the rectal vesicle to the end of this study (>2 weeks). The rise of such grains is during the terminal elimination of the granular lumenal content and mirrors the marked decrease in globular material around 8 h post-feeding (see Fig. 13). With respect to both granular and globular material there is evidence of hysteresis. I infer that this pulse of fine refractive material is the final residue of the midgut’s intracellular digestion (i.e. absorption and catabolism) of the main tranche of prey material and appears to be stored in the rectal vesicle before voiding. ‘Hypothesis (vi)’ is confirmed in principle.

Elimination of these fine refractive grains from the gut system takes a long time. The half-life (excluding the rectal vesicle) is of the order of 4.5 days (Table 1). This matches the slow final elimination of original granular prey material suggesting a rate limiting input step. In other words, the final breakdown of the most slowly degrading (resistive) granualr prey material imbibed determines the slowest rate of the final catabolic phase of egestion. Three to five half lives i.e. to 88–97% completion of elimination, is 13.5–22.5 days from the commencement of feeding—suggesting that the 2 week starvation period used in this study is insufficient to clear a mite’s gut system absolutely completely from the remains of a previous meal. Inspection of Fig. 5 confirms this, in that fine refractive grains were present at $t=0$ within the rectal vesicle and in the posterior dorsal right-hand posterior caecum lumen of some mites.

The fine grains in the pergamasid gut could of course be formed by extracellular lumenal digestive processes; or, could be from digestion intracellularly inside the gut tissue itself followed by movement out of the cells into the gut lumen (as in the haematophagous fowl mite *Dermanyssus gallinae*—see Lagutenko 1963). Future work examining gut epithelial cell histology may determine this. Whatever their origin, they may also take the role of an addition to the lumen to serve as a sink to bind all the by-products of digestion together and thereby facilitate their voiding. This would be like:- the several products secreted and excreted as colloidal material in the midgut lumen of ixodid ticks (Agyei et al. 1992); or, the accumulated mucoid substances in the starved oribatid *Galumna elimata* produced by ventricular cells (Hubert and Šustr 2001). Whilst extracellular formation cannot be excluded, the half-life of input for the fine refractive grains is 6.3 h (see Table 1), very close to the fast elimination half-life from the lumen for the original prey granular material of 6.9 h. The latter is ascribed above to be a possible intracellular process of uptake for digestion within the gut epithelium (and beyond). Given this matching of magnitudes, it is tempting to conclude that the production of fine refractive grains is also intracellular in synchronicity with any wider catabolic processes upon food absorption into the cells. Detailed micro-histological observation of gut epithelial cell changes may help to refute or confirm this in *P. longicornis*.

Raikhel (1978) showed that discrete ‘residual bodies’ are formed by tick cells during intracellular blood digestion. It is tempting to conclude that the fine refractive grains in *P. longicornis* are just these exocytic bodies. Liver cells ‘defecate’ in this way, discharging residual bodies into the intercellular space (Munnell and Cork 1980). Could the refractive grains destined to become faeces in *P. longicornis*, be the finely granular yellow-brown lipofuscin pigment granules comprised of lipid-containing residues arising from lysosomal digestion (Jung et al. 2007)? Interestingly, Dinsdale (1975) in the oribatid *Phthiracarus* sp. (using TEM) describes excretory material accumulating at the haemocoelic surface of the gut wall, which after endocytosis, passes through the cytoplasm of the gut cells as discrete bodies which then appear in the peritrophic layered faecal pellet within the gut lumen. He does not report this as guanine crystals. Note however, that no clearly defined peritrophic membrane or faecal pellet was seen in *P. longicornis*. Inspection of the gut cells in *P. longicornis* micro-histologically over time may help answer whether a similar process to this or to that of *D.gallinae* (Lagutenko 1963) or oribatids occurs. Whether this fine refractive granular material is also comprised of released mineral/polyphosphate spherites known to be stored in arachnid (see Foelix 2011), and in other invertebrate midguts (e.g. Lipovšek et al. 2002) and in engorged tick cytoplasm (Caperucci et al. 2010) as well, remains to be critically demonstrated. If true, it is not clear why for instance calcium or phosphate should be sequestered (or lead or zinc excreted as in other invertebrates—see Ludwig and Alberti (1988) and Eisler (2007) respectively) in this way in the pergamasid’s gut towards the end of digestion. Anyway, excess, magnesium, calcium and potassium is eliminated as biocrystal concretions in insects via their Malpighian tubules (see Wessing et al. 1992). This may be true too in *P. longicornis*. Could this refractive material be a part of the colour change known in some mesostigmatids (Bruce-Oliver et al. 1995) undergoing diapause in anyway? Veerman (1992) outlines a variety of food related and non-food related gut colour differences in such phytoseiids. Whatever its origin finally is, this refractive lumenal material is visually qualitatively distinct from both the large guanine crystals found in the rectal vesicle in *P. longicornis* and distinct from those refractive crystals found in its Malpighian tubules 48 h–10 days post commencement of feeding (see Bowman 2017). This opaque lumenal material is clearly the hidden ‘egestive fraction’ sought by Bowman (2017) to explain rectal vesicle expansion around 18 h.

The location of the main peak of fine refractive grains (geometric mean $\approx 40$h from a span 8 h–6 days after the start of feeding) nicely sits between the most optimistic (17 h) to the most pessimistic (52.5 h) estimate of total feeding cycle time in Bowman (2014). This total based upon gut expansion/contraction was deemed to cover ingestion, digestion and egestion (‘hypothesis (v)’). In fact 52.5 h matches 75% of the AUC for refractive grains showing that this assumption is fair—the remaining 25% of AUC being when the steady long elimination of these refractive grains is well underway (see Fig. 13). Whilst the required dynamics to confirm ‘hypothesis (v) are seen, Bowman (2014) using gut expansion/contraction may actually have slightly underestimated the contribution of egestion to the total feeding cycle time. Inspection of gut cells microhistologically could confirm or refute this—if cells at this late point are still showing intracellular digestion or evidence of continuing egestive release then the total feeding cycle time must be greater than 52.5 h in *P. longicornis*. At this point the inconsistency with the result of Reichle and Crossley (1965) remains unresolved.

**Fig. 16:**
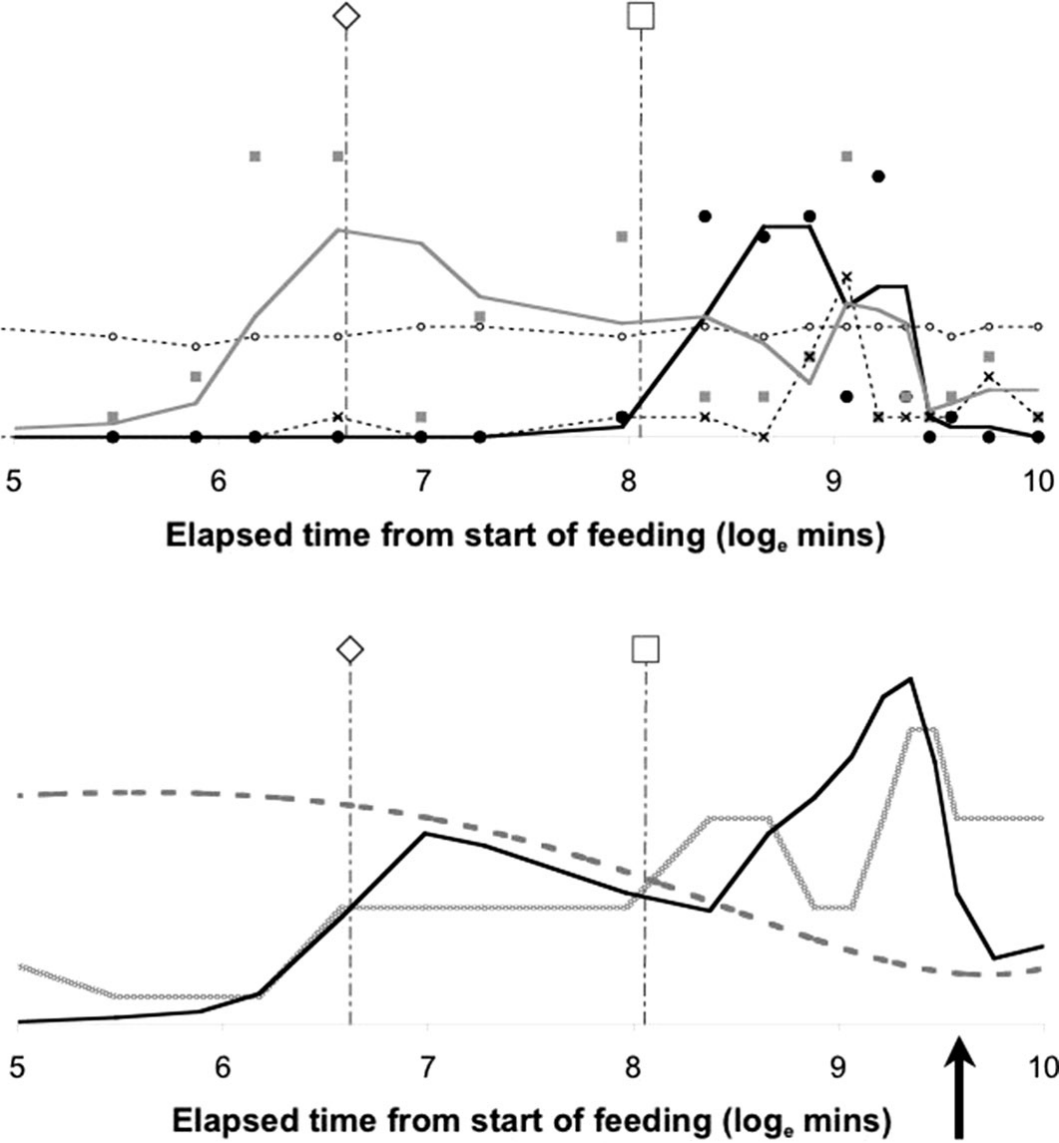
Schematics of *Pergamasus longicornis* in later stages of gut emptying/contracting. *Symbols* as in Fig. 13. *Y axis* arbitrary—data re-scaled for clear comparisons of this study’s data and that from Bowman (2017). *Upper* Lumenal guanine mean score in rectal vesicle—*small circles* and *dotted data line*—showing continual presence of guanine crystals in rectal vesicle. Overall Malpighian tubule guanine mean score—*large closed circles* and heavy solid two point moving average smooth trend—showing late peak. Refractive granular lumenal contents mean score—*grey solid squares* and *grey solid* (third order moving average) trend line—showing early rise; Membranous lumenal contents mean score—x’s and *light dotted data points line* basally, present terminally. *Lower* *Black solid line* (3rd order moving average smooth trend) = $\sum$ [ refractive grains (previous time) + Malpighian tubule guanine crystals (previous time) + membranous material (previous time) ] – showing positive association with rectal vesicle expansion/contraction mean score (see data in Bowman 2014) *grey solid* third order moving average smooth trend. Previous egestive and excretory material production drives rectal vesicle expansion. *Dashed line* indicates rescaled comparative overall gut expansion/contraction smooth trend line for Bowman (2014)—note 180$^{o}$ out of phase allowing rectal vesicle to expand. *Arrow* indicates time starvation commences from Bowman (2017)

What else could these opaque refractive grains be? Perhaps it may be, given the time offset in their peak occurrence, that the refractive grains in the gut are the actual precursors of the later large guanine crystals reported by Bowman (2017) in the rectal vesicle, rather than the latter arising from the Malpighian tubules? However, this would not explain how the Malpighian tubular guanine itself was lost from the idiosoma. Rather than indirect evidence of catabolites possibly leading to guanine, the pattern of appearance and disappearance of the fine refractive material (see Fig. 16) better fits the hypothesis of a digestive or pre-excretory product (a ‘hidden catabolic mechanism’ or surrogate ‘partly assimilated fraction’) at least arising from synchronous common catabolic processes across different body compartments. In that way they functionally match the mucoid material in starving galumnids (see Hubert and Šustr 2001).

**Fig. 17:**
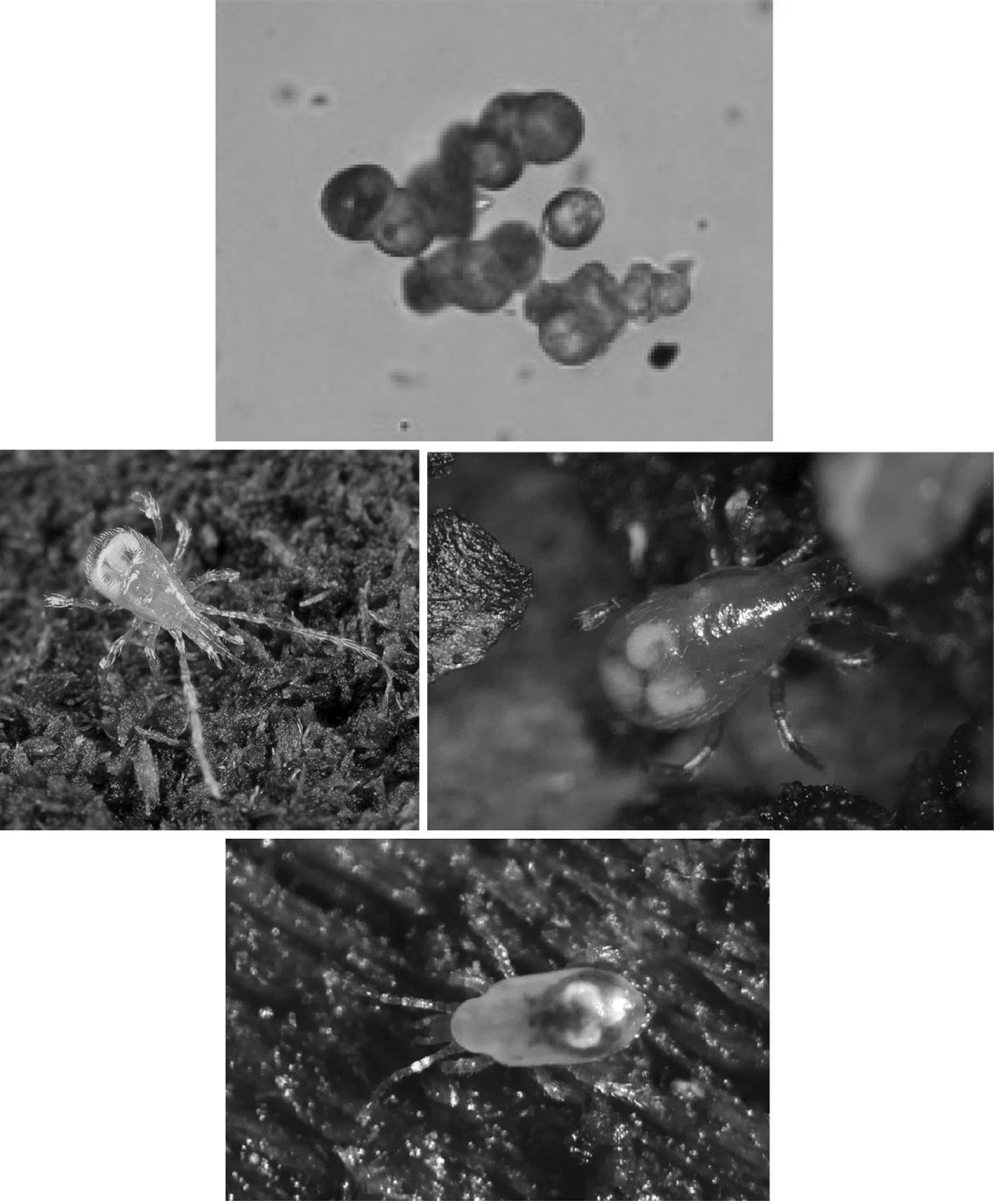
Contrasting darker egestive crystals (=xanthine?) to brilliant white excretory guanine deposits in mesostigmatids. *Upper* Xanthine uroliths from urine sediment of a dog with hereditary xanthinuria ©Minnesota Urolith Center, University of Minnesota for reference. Note mildly refractive/birefringent (opaque dull olive colour in real-life). *Middle* Soil parasitids showing gleaming pearly white Malpighian tubule crystals only (see Bowman 2017). *Left* From a colour photograph:- Norfolk, Virginia, USA 27 December 2008 ©Scott Justis with permission. *Right* From a colour photograph ©Lennart Bendixen with permission. Note guanine in rectal vesicle pygidialy. *Lower* Soil mesostigmatid showing how easy it it is to visually discern dark gut contents (egestive xanthine?) from Malpighian tubule guanine. From a colour photograph:- 29 October 2015, Germany, Schleswig-Holstein, Mohrkirch garden, Brachefläche, Holzstapel ©Lennart Bendixen with permission, where the midgut is seen to be drab olive/brown

**Fig. 18:**
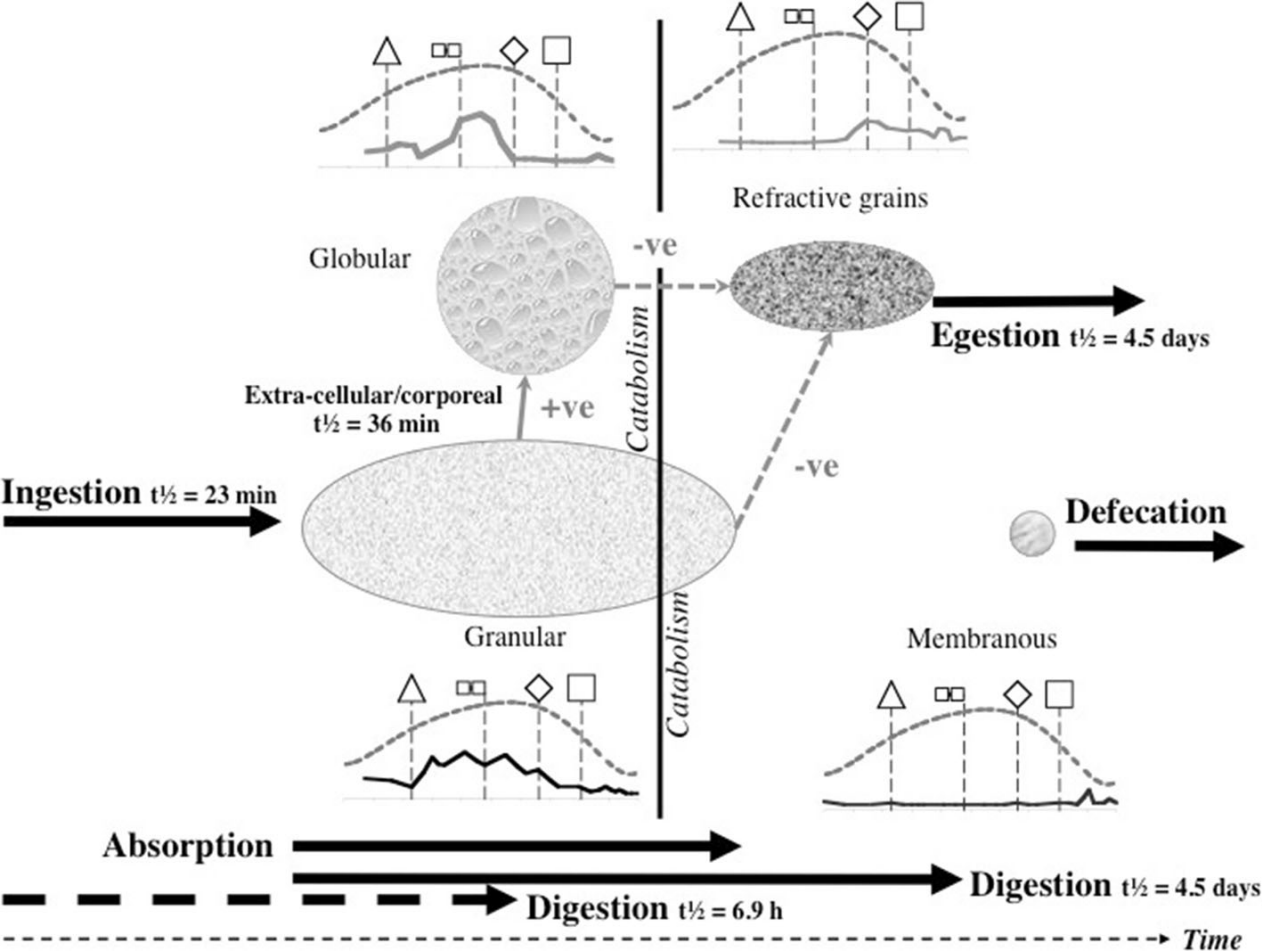
Overall summary of gut lumenal content changes in *Pergamasus longicornis* after the commencement of feeding on larval dipteran prey showing typical material classified as in this paper. Main processes with their estimated halflives shown as *strong black arrows* (*dashed arrow* represents extra-corporeal or extracellular digestion rather than intracellular digestion). Gut expansion/contraction and lumen are both positively correlated with the appearance of granular material. *Grey arrows* indicate correlated digestive states (positive or negative) and *arrow heads* the putative conversions with their half-lives. Membraneous material may be faeces or precursors to a peritrophic membrane (see “Discussion”). The pulse of imbibed pale granular material is transformed by digestion into globular material, then catabolised and lost. Later darker refractive grains appear which are possibly voided from the gut together presumably with any produced faecal material. *Inset figures* show pulse of each material over time together with overall gut expansion/contraction score from Bowman (2014). *Symbols* as in Fig. 13. The relative size and ordering *left* to *right* reflects the ordering and persistence of each lumenal content type

Perhaps the refractive grains are urate spherulites like those found in the human disease ‘gout’ (Dieppe et al. 1983)? Spherules of uric acid are known in cockroaches (Mullins 1979). Histologically, uric acid could be tested for in future work by staining with alaun haematoxylin (see Romeis 1948). However, it is not clear how uricotelic *P. longicornis* is in the wild. Perhaps, could the grains better be, in fact, the purine xanthine? Tick excreta/faeces are known to contain along with guanine an unidentified purine (along with albumin, haematin, other proteins etc)—see Obenchain and Galun (2013). Xanthine is known in the excreta of chelicerates (Polis 1990). Its concretions are less refractive and less birefringent than guanine (just as in the histological sections in this study). Xanthine inclusions can be darker in colour in practice—like a dull olive shade under a light microscope. Figure 17 shows just such dark olive material (in the colour photograph version) inside the rectal vesicle of a mesostigmatid—being quite distinct from the pearly white Malpighian tubule guanine. This fits with the proposal from other oblique evidence in Bowman (2017), that rather than being undigested prey material—idiosomal darkness in pergamasids could be indicative of egestion. Xanthine catabolically derived from ingested prey material in free-living pergamasids would then take on the role of the terminal erythrocyte degradation product haematin that is found in haematophagous acarines like dermanyssids. Enzymic confirmation or other biochemical characterisations (e.g. see Vischer and Chargaff 1948; Yokota and Shoemaker 1981) awaits future work (Fig. 18).

Also against the idea that egestion might lead to the conversion of refractive granules into more guanine finally in the rectal vesicle, would be if both materials were voided together from the mite’s anus rather than just guanine expelled. In other mites, faeces are a variety of colours—not just white indicating being only guanine. Pest control workers describe the faeces of *Dermanyssus gallinae*—the poultry red mite—as “grey ash”. Could this contain the black haematin seen in that blood feeding mite’s midgut? Yet inspection of beehives show *Varroa* spp. mites only leaving white faecal depositis. For sure in the Prostigmata, tetranychid mites produce black faeces (Hoy 2011) with other biological components in them (see Santamara et al. 2015). Further detailed studies of defecation and faecal contents in pergamasids are needed. If only guanine is ever expelled from the idiosoma, then conversion of refractive grains to guanine and admixture with Malpighian guanine crystals must occur in the rectal vesicle. If both refractive grains and guanine are expelled (even if at slightly different times), then one does not lead to the other sequentially but they represent two distinct processes with a common catabolic origin—Malpighian guanine excretion versus gut refractive grain-forming egestion. Guanine production, although synchronous with gut egestive catabolism, would then only be via a catabolic pathway through the haemocoel compartment. What happens biochemically in the haemocoel (Wyatt 1975) during feeding and digestion in *P. longicornis* thus also needs investigation.

**Fig. 19:**
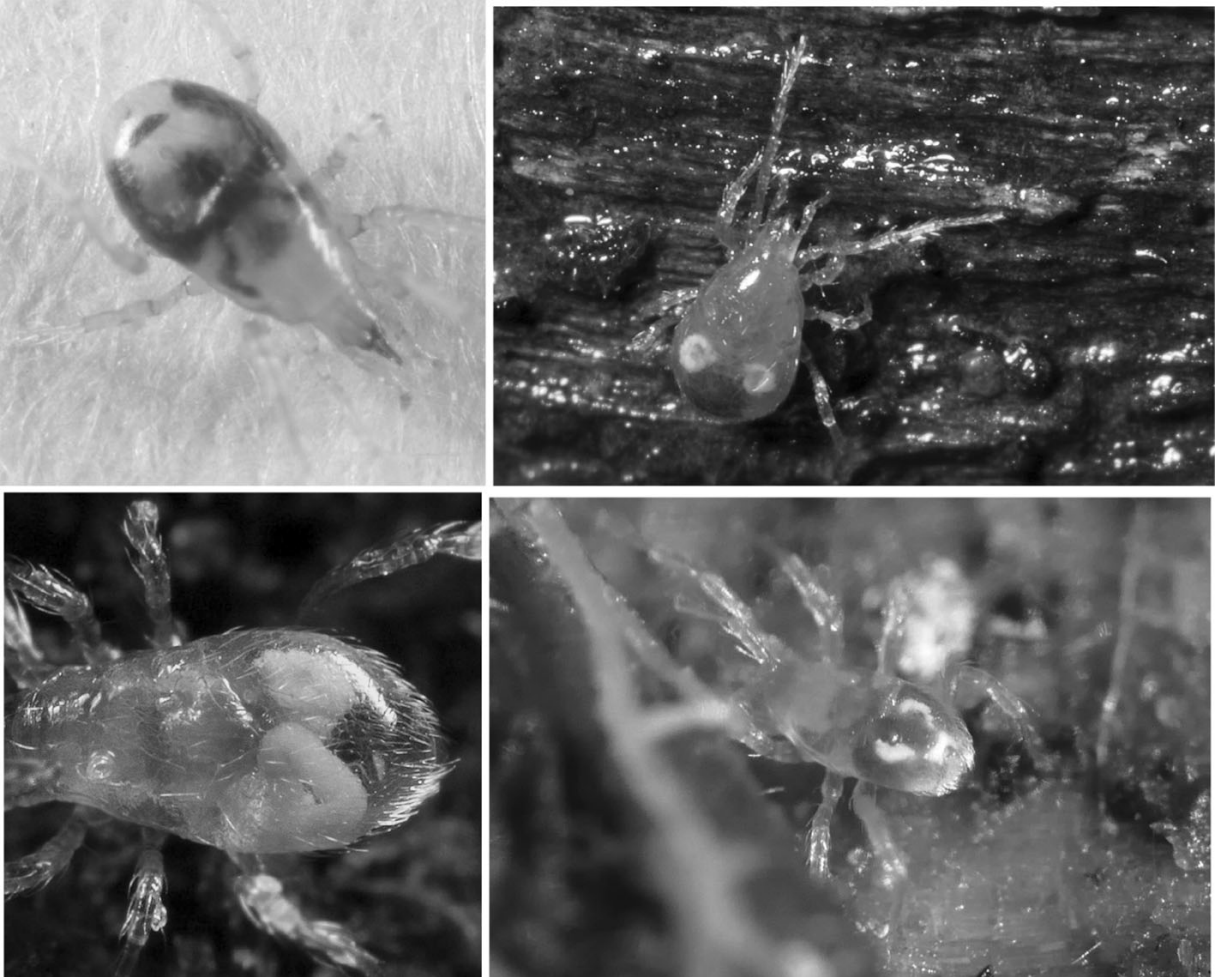
Illustrative field corroboration—nymphal mesostigmatids showing each physiological stage during the later phases of prey digestion. Sequentially from *upper left* anti-clockwise, the schema uses the equivalent time course of *Pergamasus longicornis* shown in upper frame of Fig. 13 (and data from Bowman 2017). Dark ‘less refractive’ egestive grains form in the gut and are lost from the idiosoma via the rectal vesicle. Delayed in peak but overlapping, Malpighian tubule guanine crystals form and are also lost from the idiosoma. Anti-clockwise from *upper left* these independent images represent:- egestion; excretion; egestion+excretion ( $\equiv$ early defecation); late defecation. *Upper Left* Parasitid showing dark colouration throughout the gut. This must in part be egestive material as note, importantly, the beginnings of its accumulation in the rectal vesicle (dark crescent shape pygidially to *top left*, between the dark postero-lateral caeca). Observe also the small hind-gut ‘shadow’ between the dark central posterior mesenteron of mid-gut and the rectal vesicle. There is no appreciable guanine formed in the Malpighian tubules yet at this point in time. Matches *P. longicornis* observations at $log_{e}$ values of about 5.5–8 ($\equiv$4 h–2 days after start of feeding). From a colour photograph:- Topsham, Orange County, Vermont, USA 13 November 2007 ©Tom Murray with permission where the midgut is seen to be *coloured dark brown/black*. *Lower Left* Parasitid showing empty rectal vesicle bracketed by Malpighian tubules replete with pearly white guanine. Note empty caeca now too. Matches *P. longicornis* observations at $log_{e}$ values of 8.0–9.3 ($\equiv$2–8 days after start of feeding). From a colour photograph:- Norfolk, Virginia, USA 27 December 2008 ©Scott Justis with permission. *Lower Right*: Mesostigmatid showing rectal vesicle and Malpighian tubules containing small amount of white crystals. Note dull egestive shadow over caeca. Matches *P. longicornis* observations at $log_{e}$ values of 9.3–9.6 ($\equiv$8–10 days after start of feeding, n.b. closer to 8 than 10). From a colour photograph:- 29 October 2015, Germany, Schleswig-Holstein, Mohrkirch garden, Brachfläche, Totholzhaufen ©Lennart Bendixen with permission where the midgut is seen to be *dull brown* in *colour*. *Upper Right* Parasitid mite showing posterior dark material in rectal vesicle only. Nothing anywhere in the midgut now—so this is egestive material, just like the dense black haematin in rectal vesicle of *Dermanyssus gallinae* (see Pritchard et al. 2015) and must be late on in the catabolic sequence of lumenal prey material. With the Malpighian tubules glistening with strongly refractive crystals either side, it matches *P. longicornis* observations at $log_{e}$ values of 9.3–9.6 ($\equiv$8–10 days after start of feeding, n.b. closer to 10 than 8). From a colour photograph:- Talant, France 16 February 2014 ©Christophe Quintin with permission where the rectal vesicle is seen to be *dark olive/brown* in *colour*

Notwithstanding the corroborative evidence—from field observations in Fig. 19—and the detail of possible chemical interconversion options, the common catabolic origin (assuming a well-stirred instantaneous equilibrium of the haemocoelic compartment) in the system model used by Bowman (2017)—is fairly well supported by the fact that the half-life of refractive grain input (representing *E* in the system model) at 6.3 h is close to the inferred half-life of Malpighian guanine input of 7.8 h (representing *C* in the system). Catabolism of a pulse of food does indeed seem to be a common process in *P. longicornis*—whether that via gut epithelial intracellular formation of residual bodies/xanthine concretions or via the slightly delayed haemocoelically translated Malpighian tubule excretion of guanine crystals.

The shortening of their input half-life to 2.0–2.3 h (see Table 1) and resultant steepening of their rise, if no simultaneous elimination of refractive crystals was occurring early on in digestion, could indicate a potential cascade behind the origin of this material i.e. some is driven from initial easily assimilable ingested material, some is from labile extracellularly derived material, some is from first ingested material, some is from harder to digest material etc all leading via interconversions to the same egestive waste. All of this could be dependent upon a multi-enzyme network much as in ticks.

### Membraneous and other

A sensitivity analysis of the Bayesian estimation of the change point for fragmentary membraneous material in the gut lumen indicates that the overall change point is at 1440 min ($\equiv$24 h after the commencement of feeding). This marks the beginning of an increase in the presence of sporadic disassembled membranous material in the gut lumen. This may represent terminal ‘faeces’ from a meal (current or previous) or perhaps peritrophic membrane precursors? However, note also that such erratic fragmentary membranous material is present in the gut lumen of starved mites (i.e. at $t=0$).

No kinetic modelling of the occurence of membraneous material in the gut was attempted given the paucity of the data. The late appearance (log$_{e}$(time) = 9$^{+}$ i.e. 5–6 days), after the peak of Malpighian tubule guanine production (Bowman 2017) or around the end of digestion and commencement of starvation (Bowman 2017)); the modest amounts; and, the predominance of fragmentary membranous lumenal material in the rectal vesicle, all point to this being some terminal product of feeding and digestion (cf. ‘true faeces’?) rather than evidence of the terminal formation of a peritrophic membrane. The membraneous material was not seen to be formed into a recognisable object such as a ‘faecal ball’, nor did Bowman (1984) observe anal deposition of anything other than droplets (containing white granular material—guanine crystals?) onto surfaces by *P. longicornis*.

Given that only soluble material appears to be imbibed by *P. longicornis*, one would only expect small amounts of indigestible or undigested residues (unlike in saprophagous acarines—see Prasse 1967) left as say odd bits of membranes. Teleologically for a fluid feeder like *P. longicornis*, there appears no need for a protective peritrophic membrane in this predatory mesostigmatid (unlike in ascids—see Houck 1993). Rather the fact that the only other occurrence of fragmentary membraneous material being in the lumen of starved mites, supports the premise that this is simply residual indigestible prey residues that passed any ingestive filter in that pergamasid’s mouthparts that was sampled for that time point. However, further biochemical characterisation of this membraneous material is needed to be sure of its nature as it could be a degenerative product on *bona fide* cellular death by starvation. It is not clear how this is voided as only little amounts of such membraneous material is found sporadically anywhere in the gut on commencement of feeding (see Fig. 6). Rather, the final state after feeding is actually one of an empty gut with no lumen and empty Malpighian tubules—thereafter fasting/starvation commencing in the presence of an expanded rectal vesicle (see Bowman (2017). Quite what these membrane fragments are remains a mystery.

There was too little data to speculate what the lumenal material classified as ‘Other’ might be as it appears essentially only to occur at a single time point (2 h after start of feeding). It was neither granular, nor globular nor refractive but inconsistent in nature and multi-formed. It could represent a fixation artefact or possibly evidence of an infection in that particular mite. No Bayesian estimation of a change point or sensitivity analysis was carried out due to paucity of data scoring positive for Other. No kinetic modelling is attempted given the paucity of data. Importantly, the fact that it again occurs late on in the rectal vesicle (at the same point as isolated granular and globular material is scored) suggest that all of these are artifactual or evidence of something pathological or unusual happening in those two mites sampled for those time points (see Fig. 7).

## Conclusion and diagnostic use in the field

Taking the above arguments and discussions together for *Pergamsus longicornis*, in summary (see “Introduction”):-

- Hypothesis ‘(ii)’ i.e. digestion triggered on maximum gut expansion appears to be more supported than hypothesis ‘(i)’ or hypothesis ‘(iii)’.
- ‘Hypothesis (iv)’ i.e. an anterior/posterior axis in the gut is confirmed.
- ‘Hypothesis (vii)’ regarding membraneous material as faeces is confirmed.
- There is partial support for:-
  - ‘Hypothesis (v)’ i.e. run-away catabolism.
  - ‘Hypothesis (vi)’ i.e. an egestive intermediate.
  - ‘Hypothesis (viii)’ i.e. the end point of the process.
- ‘Hypothesis (vi)’, the appropriate dynamics were detected but the extrapolation of particular cycle times (like that needed for ‘hypothesis (viii)’) has been a little underestimated to date.

Bowman (2014) proposed three phases of digestion in *Pergamasus longicornis* during and after feeding:—rapid gut filling by prey fluid imbibition (with concomitant salivary processes); continued gut filling by imbibition with the concentration of gut contents through fluid loss via coxal droplets; slow gut emptying through prey digestion. The pattern of changes in lumenal granular and globular contents in this study:- agrees with the timings of this third phase and is consilient with digestive half-lives in other mesostigmatids and ticks. This pattern further characterises the fourth phase described by Bowman (2017) as that of:- catabolic, excretory and voiding processes. The changes in the refractive granular lumenal contents described herein match the first element of this fourth phase. Bowman (2017) provided evidence of the second element. The pre-excretory egestive product present in the gut described herein explains the disparity between the end of gut contraction at 52.5 h (see Bowman 2014) and the end of the presence of any lumenal contents (see Fig. 13). There are lumenal contents way after the gut has contracted and after the worse case estimate of the feeding cycle time simply because the contracted gut retains a lumen holding some refractive grains (see Fig. 5) and ‘faecal’ membranous material (see Fig. 6). This phase (>2$^{+}$ days post-feeding) marks egestion and final defecation.

Voiding processes in *P. longicornis* still need to be investigated in detail. Nevertheless, Fig. 16 nicely illustrates that for a single larval dipteran prey meal, there is a strong correlation between rectal vesicle size at any one time (*t*) and the sum of the probable inputs from the preceding time point (i.e. $\sum$ ([lumenal refractive grains$_{(t-1)}$] + [lumenal membraneous material$_{(t-1)}$] + [Malpighian tubule guanine crystals$_{(t-1)}$] ). Excretory and faecal production as a pulse clearly drives rectal vesicle expansion. The egestive phase between commencement of gut emptying at 12.5 h after the start of feeding and the 52.5 h time point is marked by the gut contracting in size (as Bowman (2014) described) but with its lumen being packed with putative catabolites. From 52.5 h post the start of feeding onwards is then proper excretion and defecation. The gut finally empties, continues to contract in magnitude and eventually loses its lumen after an extended period. The catabolites pass into the rectal vesicle during the period of Malpighian tubule excreta production (see Bowman 2017) and are voided via the rectal vesicle (see field examples in Fig. 19). Membraneous material is the last to persist (see Fig. 6). However, the actual final physical voiding mechanisms of any material mechanically out of the idiosoma remain obscure.

Figure 18 formalises the results of this study. Confirmation of half-lives and how lumenal material is intracellularly processed awaits micro-histological examination of gut cells over time. Whether the gut epithelium behaves rhythmically over time as reported in ixodid ticks (Grigor’eva 2003, 2005, 2006)—which Fig. 13 *Lower* might suggest—awaits organ culturing to verify. An artifactual pattern like this could also result from the between mite stochastic variation arising due to the destructive sampling method used in this study. Or, it could be evidence of midgut peristalsis—known to move lumenal cells and material during defecation in ticks (Sonenshine and Roe 1991)—which has been frozen in time by the histological fixation of the mite at that juncture. Similarly how reproductive demands may change digestive activity (as in ticks—see Mendiola et al. 1997) remains to be investigated in *P. longicornis*. The catabolic interconversions producing the final fine refractive grains also require biochemical tracking over time in a follow-up study. Whether refractive grains convert into guanine or not rectally is not entirely clear. Similarly if their catabolic production is strictly synchronised with guanine shipped out from the haemocoel into the Malpighian tubules awaits confirmation.

Are refractive grains dark to the eye for an ecologist to see? Indeed most probably. Certainly they are darker and more opaque under light microscopy in histological sections than the original non-refractive granular larval dipteran prey material first imbibed. In fact, the latter needed to be stained to be seen well. Most prey soil insect larvae are pale white/colourless, as are nematodes and many (but not all) collembola. Prey acarine tissues themselves are pale/colourless. A pale replete gut should thus infer ingestion/digestion. Unless *P. longicornis* specialised just in feeding upon say purple coloured springtails like *Hypogastrura* sp. or *Tetrodontophora* sp. (and there is no evidence that they do), the bulk of prey material imbibed should be colourless. Figure 1 nicely shows mesostigmatids in the field with clear or gently pale opaque idiosomas matching the proposed unfed and early fed status of *P. longicornis*. Unfortunately, the cuticle of *P. longicornis* is too sclerotised to see how pale the gut is by eye. Colour photographs of adult pergamasids showing white Malpighian tubule guanine and dark ‘xanthine’ deposits unfortunately also do not make good black and white illustrations due to their dark integument (see for instance Fig. 1 in Bowman (2014)). However, if the opaque refractive grains are dark—as the coherent scheme of this paper would indicate—Fig. 19 shows how visual consilience of the results of this study with observations in the field of other paler integument mesostigmatids. Dark opaque midgut material must be egestive here as it appears in the rectal vesicle in appreciable amounts (initially imbibed non-refractive grains generally do not—compare distribution across regions in Fig. 3 versus those in Fig. 5). Dark opaque material must be egestive as it co-occurrs with substantial guanine amounts in the Malpighian tubules (initially imbibed non-refractive grains do not—see Figs. 13 and 19). The physiological story in these independent photographs is compelling—but confirmation say by extraction of the midgut refractive granular material in *P. longicornis* and spectrophotometric testing would help finally determine that it is dark.

**Fig. 20:**
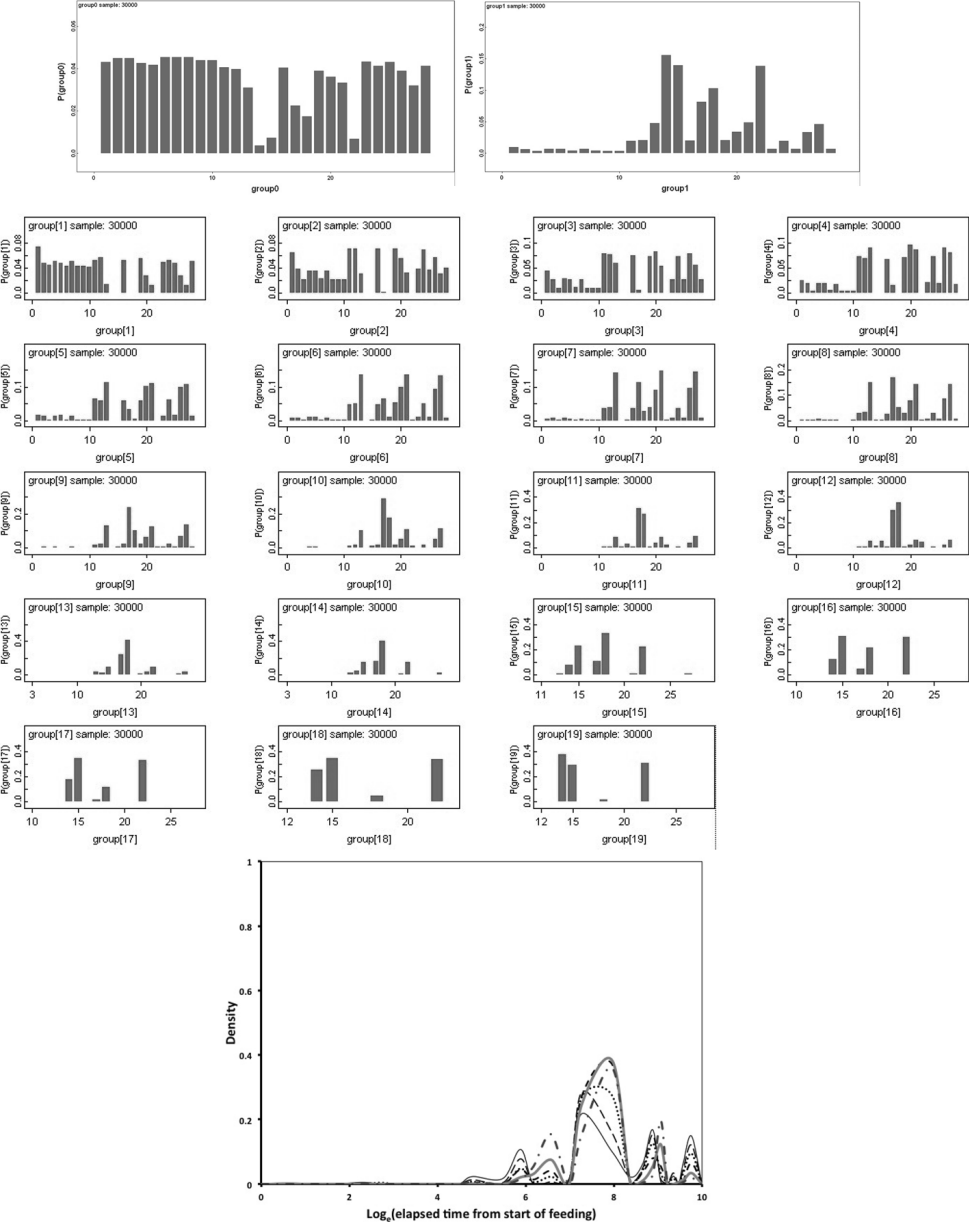
Posterior predictive distributions of likely time since the start of feeding based upon the results in this paper for use by ecologists in the field collecting *Pergamsus longicornis* and scoring the presence of refractive dark material in the midgut. *Upper Left* Given a single mite showing midgut refractive grains. *Upper Right* Given a single mite does not show midgut dark refractive grains. Note very small scale to *left hand side*—absence of dark material is not very informative. *Middle Block of small histograms* Given a number of *P. longicornis* mites (out of 20) showing midgut dark material in a population sample. Ranges from 1 out of 20 for *top left small panel* to 19 out of 20 for *bottom right small panel*. *Lower* Nomogram of posterior predictive density for 9–14 mites out of 20 showing dark refractive grains in midgut in an ecological sample versus log$_e$ time in min after the start of feeding. 9 mites = *thin solid line*, 10 = *long dash line*, 11 = *dotted line*, 12 = *dashed line*, 13 = *solid thick line*, 14 = *bold dot-dashed line*. Note that about 12 mites scoring positive for dark refractive material in the midgut out of 20 infers a 4 out of 10 chance that the typical time from start of feeding in that population sample was about 1.9 days ago (i.e. around time index = 18). Any larger proportions out of 20 indicate the same peak inferred time—spanning around 23 h–2.2 days since the start of feeding

**Fig. 21:**
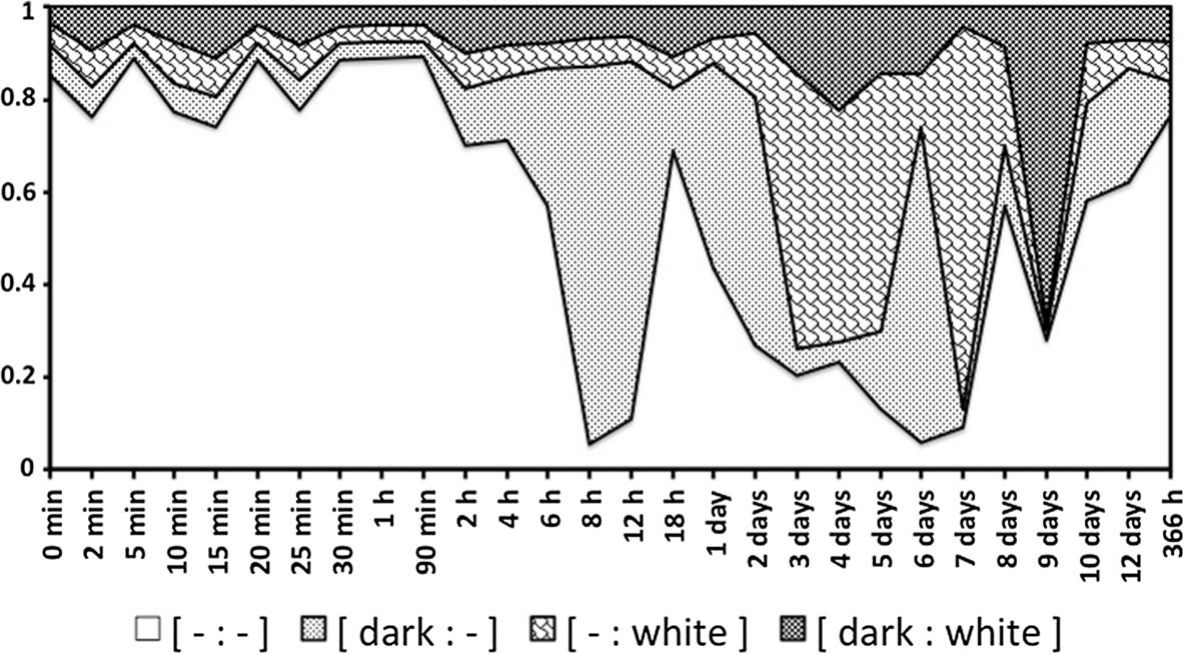
Posterior probability estimates for combination of visual physiological states versus collection times after the start of feeding in *Pergamasus longicornis*. Stacked multinomial combinations are of:- the absence or presence of opaque refractive material in the midgut from this study, with, the absence or presence of white guanine crystals in the Malpighian tubules associated with that midgut region (ex Bowman 2017). Left hand scale is probability (note:- sum of multinomial estimates = 1). Uses non-informative *Dirichelet* parameter prior with $\alpha \ concentration=1$. In legend ‘-’ means absent. *Note* high density of [-:-] state early on (=ingestion/digestion); then transition through state [ dark refractive grains:-] (=egestion); and then onto [-: white guanine crystals ] (=excretion); to finally [dark:white ] (=defecation)

**Fig. 22:**
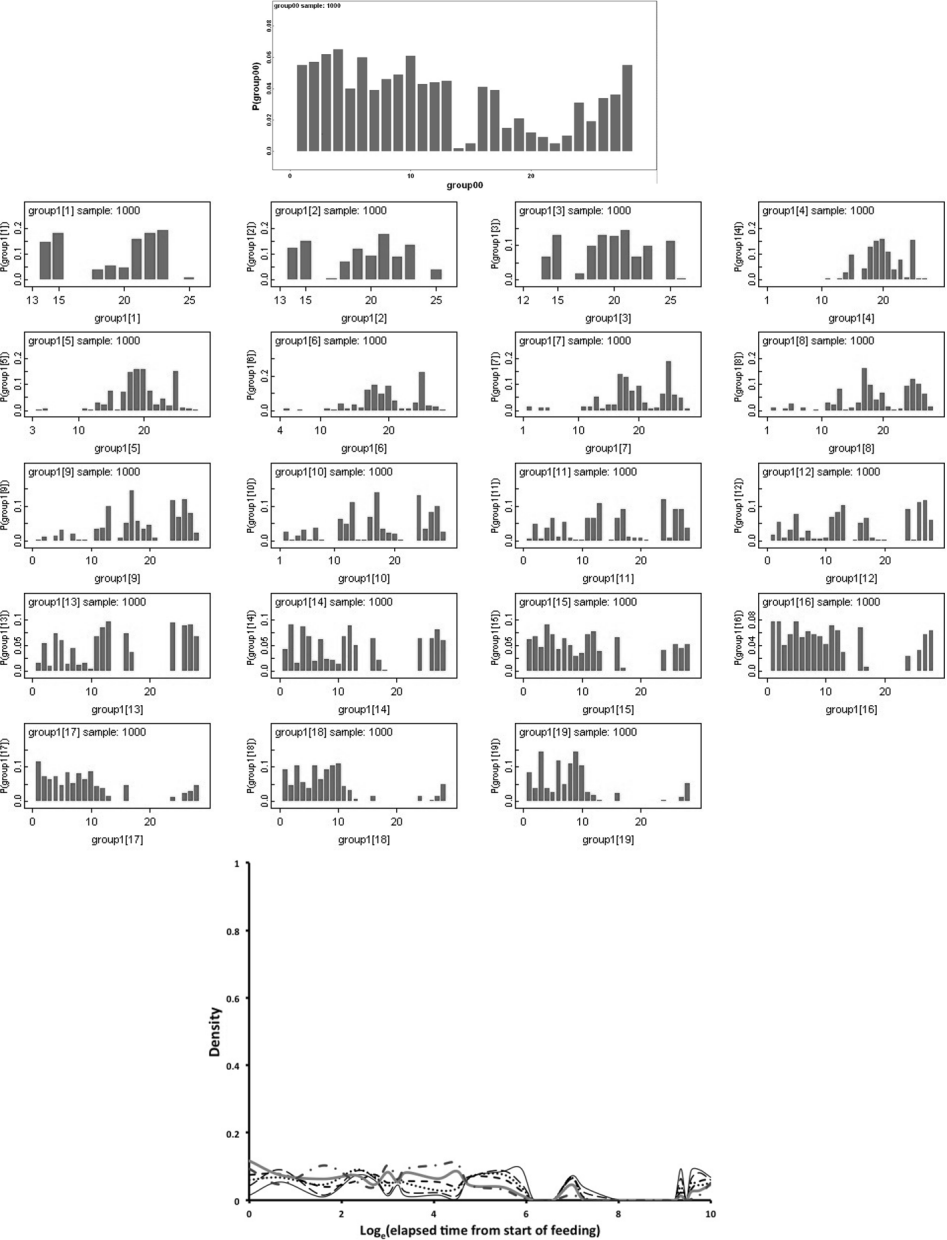
Posterior predictive distributions of likely time since the start of feeding based upon the results in this paper and those of Bowman (2017) for use by ecologists in the field collecting *Pergamasus longicornis* and simultaneously scoring for the lack of refractive dark material in the midgut and the lack of guanine in the Malpighian tubules ( $\equiv$ ingestion/digestion). *Upper* Given a single mite showing no midgut refractive grains and no Malpighian tubule guanine. Note very scale to *left hand side* showing mutual absence is not very informative. *Middle Block of small histograms* Given a number of *P. longicornis* mites (out of 20) showing no midgut dark material and no Malpighian tubule guanine in a population sample. Ranges from 1 out of 20 for *top left small panel* to 19 out of 20 for *bottom right small panel*. *Lower* Nomogram of posterior predictive density for 13–18 mites out of 20 showing no dark refractive grains in midgut and no Malpighian tubule guanine in an ecological sample versus log$_e$ time in min after the start of feeding. 13 mites = *thin solid line*, 14 = *long dash line*, 15 = *dotted line*, 16 = *dashed line*, 17 = *solid thick line*, 18 = *bold dot-dashed line*. Note that from 13 mites scoring negative for both dark refractive material in the midgut and negative for white Malpighian tubule guanine out of 20 infers (despite the low probability) that the typical time from start of feeding in that population sample was about 0–6 h ago (i.e. time index <13). Any larger proportions out of 20 indicate the same peak inferred time—expected value 32.3 min-3.2 h since the start of feeding. There is a possibility that such mites might be fasting/starved—see *right hand side* of density $\ge$8 days. Many mites would be needed to confidently infer either a non-fed or an early feeding status on average for field samples

**Fig. 23:**
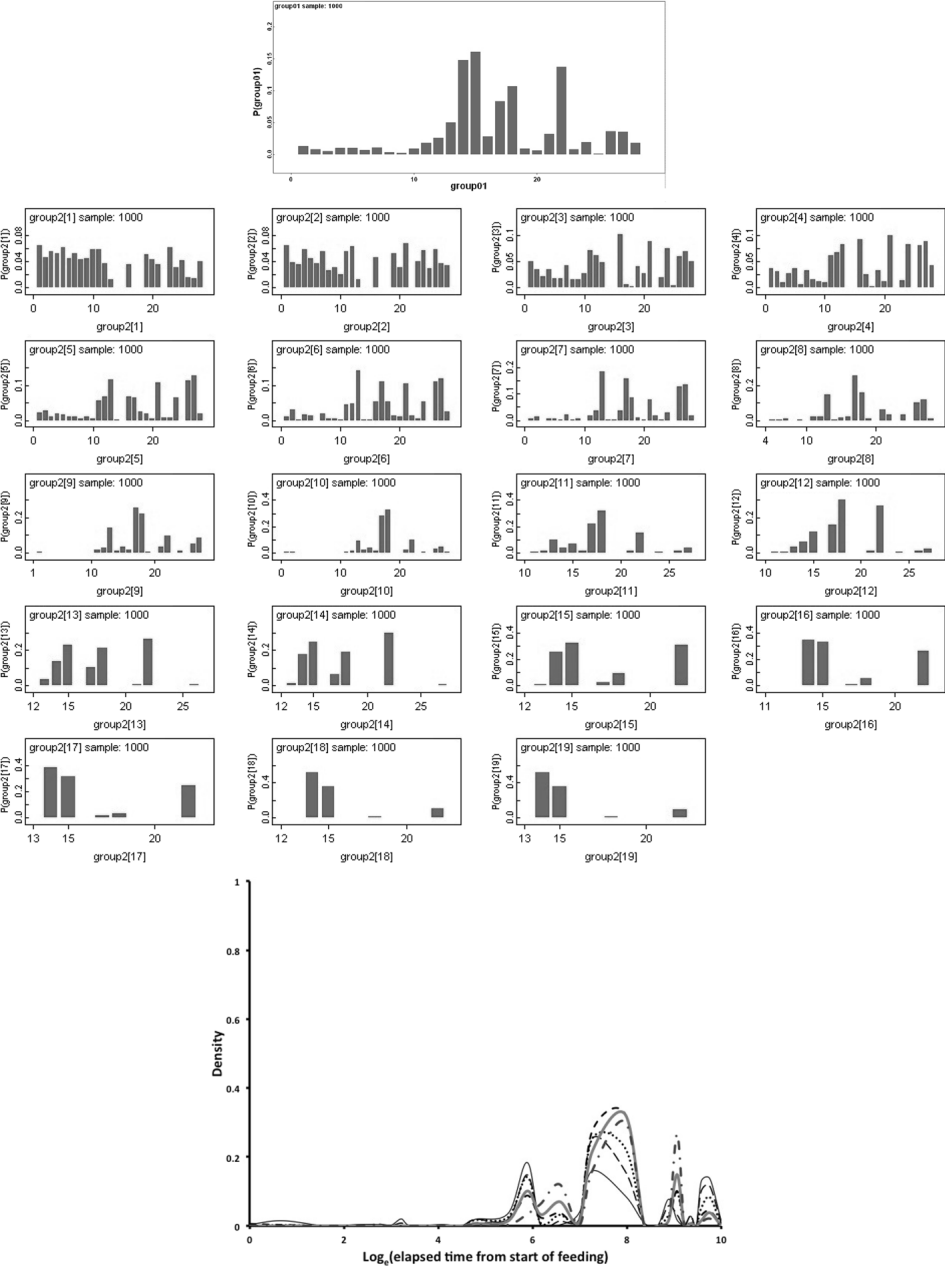
Posterior predictive distributions of likely time since the start of feeding based upon the results in this paper and those of Bowman (2017) for use by ecologists in the field collecting *Pergamasus longicornis* and simultaneously scoring for the presence of refractive dark material in the midgut but the lack of guanine in the Malpighian tubules ($\equiv$egestion). *Upper:* Given a single mite showing midgut refractive grains but no Malpighian tubule guanine. Note reasonable scale to *left hand side* but broad spread. *Middle Block of small histograms* Given a number of *P. longicornis* mites (out of 20) showing midgut dark material but no Malpighian tubule guanine in a population sample. Ranges from 1 out of 20 for *top left small panel* to 19 out of 20 for *bottom right small panel*. *Lower* Nomogram of posterior predictive density for 7–12 mites out of 20 showing dark refractive grains in midgut but no Malpighian tubule guanine in an ecological sample versus log$_e$ time in min after the start of feeding. 7 mites = *thin solid line*, 8 = *long dash line*, 9 = *dotted line*, 10 = *dashed line*, 11 = *solid thick line*, 12 = *bold dot-dashed line*. Note that about 10 mites scoring positive for dark refractive material in the midgut but negative for white Malpighian tubule guanine out of 20 infers about a 4 out of 10 chance that the typical time from start of feeding in that population sample was about 1.4 days ago (i.e. around time index = 17–18 on average). Any larger proportions out of 20 indicate the same overall peak inferred time—spanning around 12.6 h–1.8 days since the start of feeding but which becomes concentrated at its extremes (time index 14 = 8 h and 22 = 6 days)

**Fig. 24:**
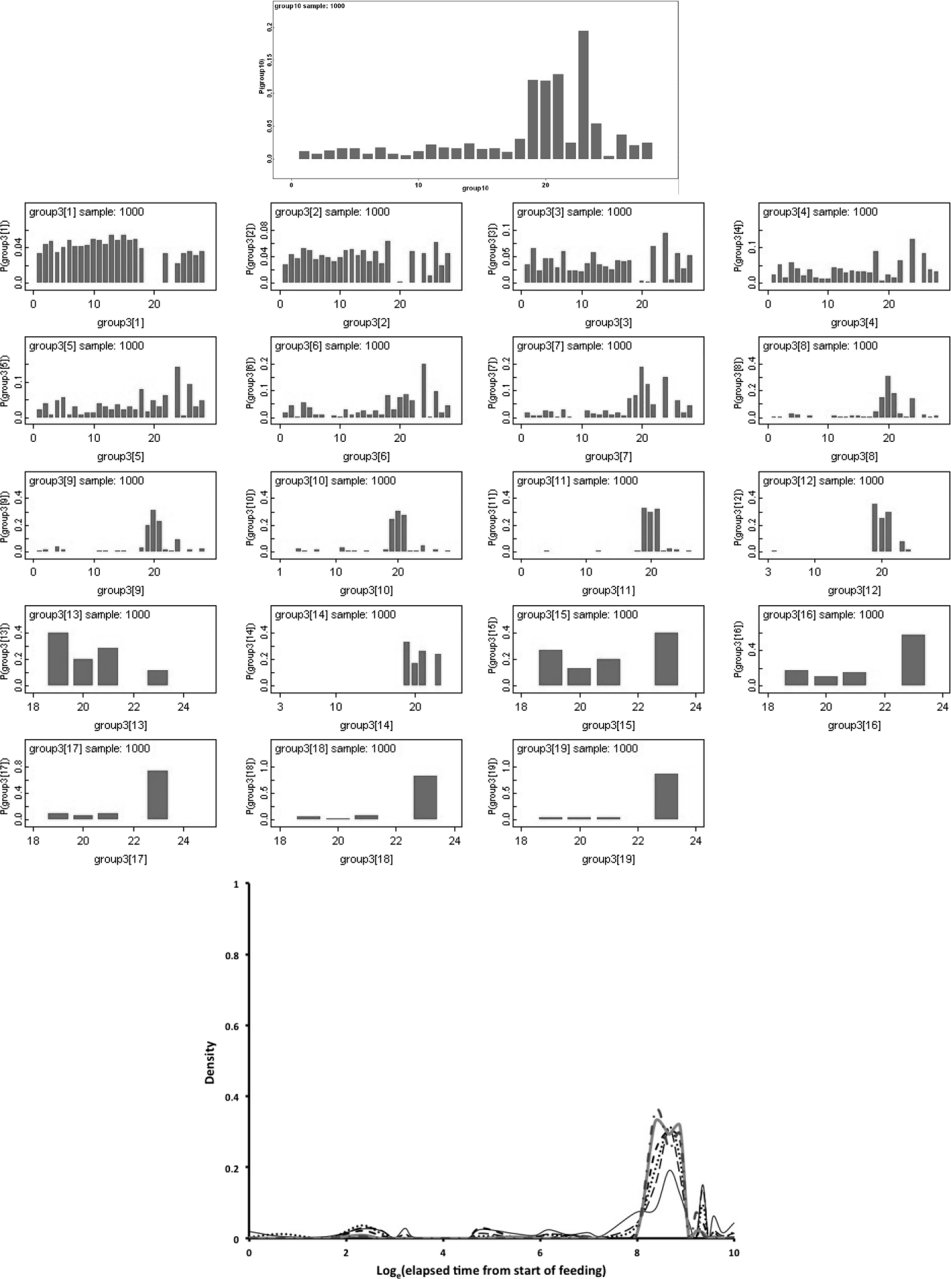
Posterior predictive distributions of likely time since the start of feeding based upon the results in this paper and those of Bowman (2017) for use by ecologists in the field collecting *Pergamasus longicornis* and simultaneously scoring for the lack of refractive dark material in the midgut but the presence of guanine in the Malpighian tubules ($\equiv$excretion). *Upper* Given a single mite showing no midgut refractive grains but showing Malpighian tubule guanine. Note reasonable scale to *left hand side* and modest spread. *Middle Block of small histograms* Given a number of *P. longicornis* mites (out of 20) showing no midgut dark material but showing Malpighian tubule guanine in a population sample. Ranges from 1 out of 20 for *top left small panel* to 19 out of 20 for *bottom right small panel*. *Lower* Nomogram of posterior predictive density for 7–12 mites out of 20 showing no dark refractive grains in midgut but showing Malpighian tubule guanine in an ecological sample versus log$_e$ time in min after the start of feeding (compare to Bowman 2017 Fig. 11 *Lower*). 7 mites = *thin solid line*, 8 = *long dash line*, 9 = *dotted line*, 10 = *dashed line*, 11 = *solid thick line*, 12 = *bold dot-dashed line*. Note that about 12 mites scoring negative for dark refractive material in the midgut but positive for white Malpighian tubule guanine out of 20 infers about 4 out of 10 chance that the typical time from start of feeding in that population sample was about 3–7 days ago (i.e. around time index = 19–23). Any larger proportions out of 20 indicate the same peak inferred time—expected value 3.9–6.5 days since the start of feeding but a domination of predictions at 7 days

**Fig. 25:**
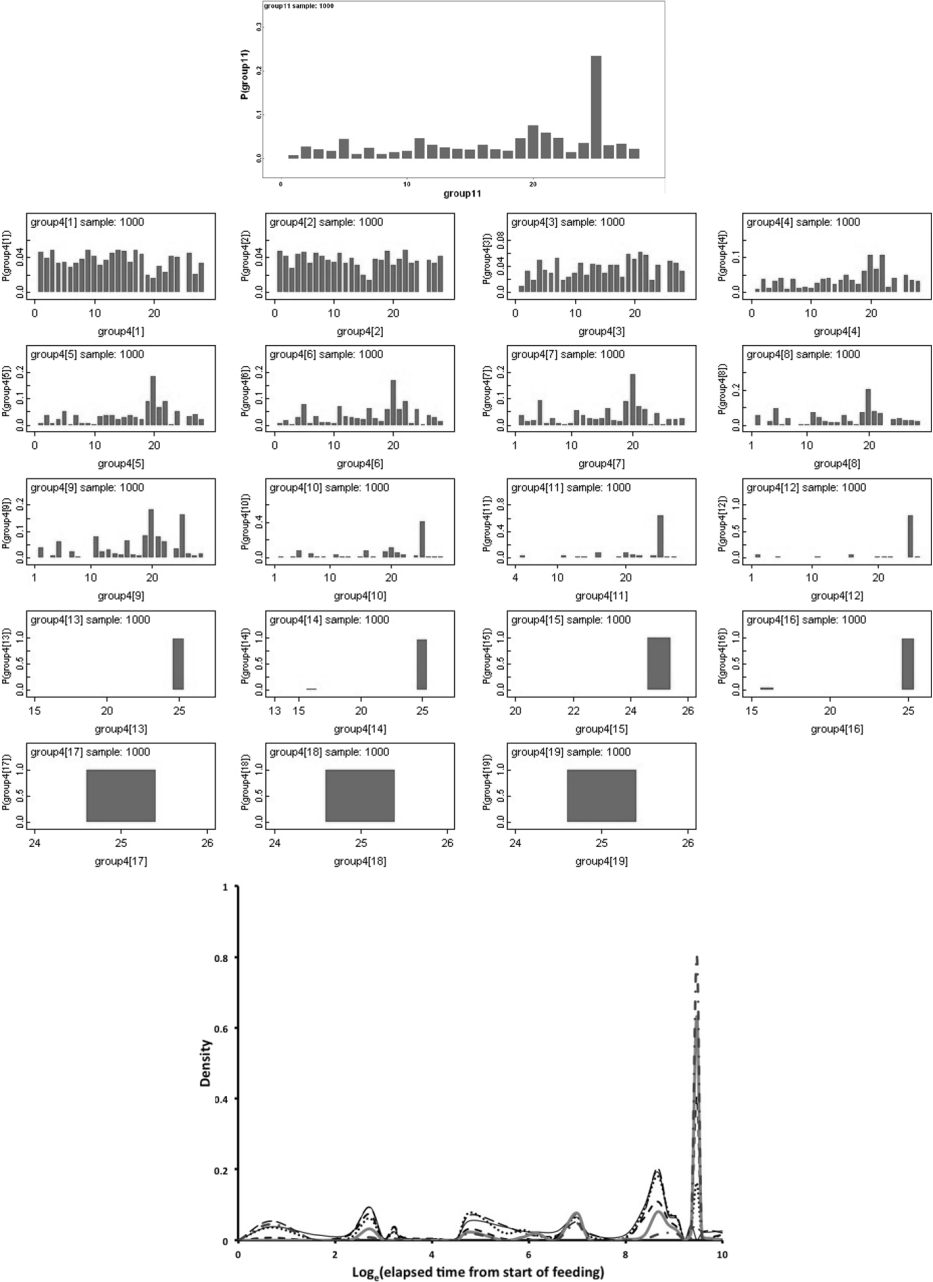
Posterior predictive distributions of likely time since the start of feeding based upon the results in this paper and those of Bowman (2017) for use by ecologists in the field collecting *Pergamasus longicornis* and simultaneously scoring for the presence of refractive dark material in the midgut and for the presence of guanine in the Malpighian tubules ($\equiv$defecation). *Upper* Given a single mite showing midgut refractive grains and Malpighian tubule guanine at the same time. Note reasonable scale to left hand side and very strong peak. *Middle Block of small histograms* Given a number of *P. longicornis* mites (out of 20) showing midgut dark material and Malpighian tubule guanine in a population sample. Ranges from 1 out of 20 for *top left small panel* to 19 out of 20 for *bottom right small panel*. *Lower* Nomogram of posterior predictive density for 7–12 mites out of 20 showing dark refractive grains in midgut and Malpighian tubule guanine in an ecological sample versus log$_e$ time in min after the start of feeding. 7 mites = *thin solid line*, 8 = *long dash line*, 9 = *dotted line*, 10 = *dashed line*, 11 = *solid thick line*, 12 = *bold dot-dashed line*. Note that about 11 mites scoring positive for both dark refractive material in the midgut and for white Malpighian tubule guanine out of 20 infers a greater than 50:50 chance that the typical time from start of feeding in that population sample was about 9 days ago (i.e. around time index = 25). Larger proportions out of 20 indicate the same peak inferred time—spanning around 8.3–8.9 days since the start of feeding with 9 day predictions predominating

What else supports the assumption (opaque refractive grains = macroscopic darkness) ? Detailed statistical argument also confirms this (and the above physiological sequencing). Posterior predictive distributions (Aitchison and Dunsmore 1975) and resultant nomograms for pergamasids showing refractive mid-gut grains are shown in Fig. 20, and also together in combination with the Malpighian tubule guanine results of Bowman (2017) in Figs. 21, 22, 23, 24 and 25. Twelve out of 20 mites collected in the field scoring for (dark) egestive refractive material in the midgut would indicate a 4/10 chance that pergamasid mites in a sample have, on average, fed 1.9 days ago. Refractive opaque midgut material is about twice as less predictive than using Malpighian tubule guanine for diagnosis (see Bowman 2017, Fig. 11) as expected from its wider peak over time. The appearance of such midgut material precedes white Malpighian tubule guanine production just as expected if darkness *was* egestive grains. Rectal vesicle guanine scoring (or rectal vesicle scoring for opaque refractive material) on their own are poorly predictive prospectively of the elapsed time from the start of feeding (*results not shown*). Further evidence for this assumption (darkness = opaque refractive grains) comes from turning to if multinomial combinations of midgut and Malpighian tubule visual scoring are consilient with the proposed scheme. Poor predictions arise from mites that neither show opaque (dark) midgut material nor associated white Malpighian tubule crystals (see Fig. 22). Scoring mites for the presence of opaque (dark) refractive midgut material but the absence of Malpighian tubular guanine (Fig. 23) confirms the result in Fig. 20, that is—about 10 mites scoring positive for opaque i.e. dark refractive material in the midgut but negative for white Malpighian tubule guanine out of 20 would infer about a 4 out of 10 chance that the typical time from start of feeding in that population sample was about 1.4 days ago. Scoring mites for no dark refractive midgut material but the presence of guanine indicates that about 12 mites scoring out of 20 would infer about 4 out of 10 chance that the typical time from start of feeding in that population sample was about 3–7 days ago (Fig. 24). This compares well with the previous result of Bowman (2017) showing inferences including egestive material remain coherent. A combination of scoring for opaque (dark) midgut contents and white Malpighian tubule guanine in as few as 10–11 mites out of 20 would strongly indicate feeding on average was 9 days ago (Fig. 25). Figure 21 shows why this relative ordering works so well and supports that refractive egestive grains seen histologically *are* the dark material sought in photographs. The time ordering emergent from the data fits.

What else supports the assumption (opaque refractive micro-grains = darkness)? If the original prey material imbibed could also be easily visually scored (not just as pale white as in Fig. 1 *Lower*)—but say by using red or green coloured prey (as in phytoseiid predators eating *Tetranychus* spp.) then such could be used to validate this result temporally (and also in itself to infer feeding status in the wild too). So consider for instance, the posterior predictive density calculated for non-refractive pale granular material first imbibed in this study arising from 13 to 18 mites out of 20 in a field sample would give about a 4 out of 10 chance that the typical time of feeding spanned 15 min–12 h with an expected value of around 51.9–76.3 min from the start of feeding (*plots not shown*). This is very close to the observed average time (56–96 min) actual feeding takes place in *P. longicornis* (Bowman 1987). That is inferred ingestion/digestion from gut scoring matches observed ingestion/extracorporeal digestion if non-refractive grains are *not* dark. Furthermore, this estimate is far too early for the imbibed material to be the dark material sought as it well precedes guanine production in time and therefore obviates any chance of seeing mites scored with both (which one does—see Figs. 17 and 19. Recall guanine is only seen late-on beyond the 52.5 h total feeding cycle time (its peak = 5 days after the start of feeding, Bowman (2017)) and at that point the original non-refractive granular prey material is minimal (see Fig. 3). Such cannot be the dark material ! A similar denial can be made regarding globular material. By eliminative argument and the temporal facies, the opaque refractive grains *are* the dark material sought. Further spectrophotometric investigation of gut changes should be done with dyed food in follow-up work and also appropriate dark refractive material sought for in phytoseiids, but I confidently conclude that dark opaque midguts in *P. longicornis* on balance indicate refractive egestive (xanthine?) grains and vice versa.

I commend the nomograms for ecologists to deploy and test out (graphs for other proportions of sampled mites can be provided on request).

Despite starvation/fasting commencing around 10 days, up to 3 weeks may be needed to fully clear the mite’s gut system from a previous meal. This marks *P. longicornis* out like many other long-living arachnids as an intermittent ‘bolus’ feeder with a slow digestion capable of enduring very long periods of starvation. Complete loss of gut lumen during this time may take 5–9 weeks since it has first fed. Together with the above results of high speed ingestion and short initial digestive half-lives—all these are undoubted adaptations to its forest soil life where prey densities are low yet instant energy demands for an active predatory lifestyle are high.
